# Disentangling eco-evolutionary dynamics of predator-prey coevolution: the case of antiphase cycles

**DOI:** 10.1038/s41598-017-17019-4

**Published:** 2017-12-07

**Authors:** Ellen van Velzen, Ursula Gaedke

**Affiliations:** Department of Ecology and Ecosystem Modelling, Institute of Biochemistry and Biology, University of Potsdam, Maulbeerallee 2, 14469 Potsdam, Germany

## Abstract

The impact of rapid predator-prey coevolution on predator-prey dynamics remains poorly understood, as previous modelling studies have given rise to contradictory conclusions and predictions. Interpreting and reconciling these contradictions has been challenging due to the inherent complexity of model dynamics, defying mathematical analysis and mechanistic understanding. We develop a new approach here, based on the Geber method for deconstructing eco-evolutionary dynamics, for gaining such understanding. We apply this approach to a co-evolutionary predator-prey model to disentangle the processes leading to either antiphase or ¼-lag cycles. Our analysis reveals how the predator-prey phase relationship is driven by the temporal synchronization between prey biomass and defense dynamics. We further show when and how prey biomass and trait dynamics become synchronized, resulting in antiphase cycles, allowing us to explain and reconcile previous modelling and empirical predictions. The successful application of our proposed approach provides an important step towards a comprehensive theory on eco-evolutionary feedbacks in predator-prey systems.

## Introduction

Evolutionary change can occur on ecological timescales^1–3^, resulting in the complex feedbacks of eco-evolutionary dynamics^4,5^. A striking number of examples have been found in traits directly involved in predation^6–9^ and defense against predators^9–12^, indicating contemporary evolution is common in predator-prey interactions, with potentially dramatic impacts on predator-prey dynamics. Rapid evolution of prey defense in response to changes in predator abundance may stabilize or destabilize dynamics^13^, or qualitatively change the shape of predator-prey cycles^10,12^. For example, rapid prey evolution may result in antiphase cycles^10,14^, where the predator lags behind the prey with half the period, rather than the ¼-lag cycles predicted by non-evolutionary models^15^. Theoretical study has demonstrated that antiphase cycles are expected when defense is highly effective^16,17^.

In strong contrast with studies on prey evolution alone, the impact of predator-prey coevolution on predator-prey dynamics remains poorly understood. Modelling studies on coevolution revealed a wide range of possible predator-prey dynamics; these include the antiphase cycles found in models on prey evolution^18,19^, but also in-phase or reversed cycles^18–21^. Moreover, predictions on when any of these dynamics should be found are contradictory: for example, antiphase cycles could only occur when predator adaptation was slow compared to ecological dynamics^22^, or slower than prey adaptation^18^, while they were found for extremely fast predator adaptation in others^20,21^. In absence of comprehensive empirical evidence, a predictive theory of how coevolution affects predator-prey dynamics is still missing.

The main challenge in developing a comprehensive theory lies in the complexity of model dynamics, with ecological and evolutionary changes on two trophic levels all interacting simultaneously. As such models are not analytically tractable, study is generally limited to numerical simulations, which to some extent form a black box yielding results that are difficult to disentangle and interpret. Previous attempts to reduce model complexity have used a separation between ecological and evolutionary timescales, assuming either that evolutionary dynamics are much slower than ecological dynamics^23^, or the reverse, that evolutionary dynamics are more rapid^17,21^. This removes real-time interactions between ecological and evolutionary dynamics, resulting in a more analytically tractable model. While important insights can be gained from such models, they inherently lack the critical point of eco-evolutionary dynamics: that ecological and evolutionary processes are fundamentally entwined and occur at the same timescales^1,5^.

Supporting this, models that integrate ecological and evolutionary timescales show that the speed of adaptation at the two trophic levels, either relative to the ecological dynamics or to each other, strongly affects eco-evolutionary dynamics^18,19,22^. Thus, gaining a mechanistic understanding of how the interaction between ecology and evolution shapes community dynamics requires maintaining the full complexity of eco-evolutionary models. At the same time, it is imperative that we move beyond descriptive analysis of their dynamics, and towards a more fundamental understanding of the underlying mechanisms generating them.

We develop a new approach to gain such fundamental understanding, by extending a well-established mathematical decomposition for deconstructing eco-evolutionary dynamics into their component effects (the Geber method^1^). Through analyzing the temporal dynamics and interactions between these components, we disentangle how changes in prey biomass, predator biomass, and prey and predator traits impact and drive predator-prey dynamics. We apply this approach to a specific question: when and how does coevolution result in antiphase cycles? For this we use a model with a single prey and predator type, each with an adaptive trait (defense for prey, offense for predators; see Methods for details). We show that coevolution may change the predator-prey phase relationship, but the predator appears to follow the *effective prey biomass* with a ¼-lag. The effective prey biomass is a measure of how much prey biomass is available to the predator, and is determined by prey biomass and trait dynamics. We use our component analysis to demonstrate how the dynamics of the effective prey biomass, and through this the predator-prey phase relationship, are determined by the interaction between between prey biomass and trait dynamics, resulting in a surprisingly simple predictive theory for phase relationships under predator-prey coevolution.

## Results

### Eco-evolutionary dynamics

We focus our analysis on two pairs of parameters that influenced the predator-prey relationship in previous models: the speed of adaptation in the prey and predator^18,19,22^ (*G*_*x*_ and *G*_*y*_, “speed analysis”); and the costliness of defense and offense^20,21^ (*c*_*x*_ and *c*_*y*_, “cost analysis”). All parameters and their values can be found in Table 1.

**Table 1:** List of parameters and parameter values.

Parameter	Description	Value	
		Speed analysis	Cost analysis
*K*	carrying capacity prey	**1.0**, 1.5, 2.0	**1.0**, 1.5, 2.0
*r*_0_	maximum growth rate prey	**1.0**	**1.0**
*g*_0_	maximum conversion efficiency	**1.0**	**1.0**
*θ*	efficiency of defense	**10**	**10**
*d*	per capita mortality predator	**0.1**	**0.1**
*a*_0_	maximum attack rate	**1.0**	0.5, 0.75, **1.0**, 1.25, 1.5, 1.75, 2.0
*h*	handling time	**0.1**, 1.0	**1.0**, 1.5, 2.0
*c*_*x*_	costliness of defense	2.0, **3.0**, 4.0, 5.0	*0–10*
*c*_*y*_	costliness of offense	**2.0**, 3.0, 4.0, 5.0	*0–10*
*G*_*x*_	speed of adaptation prey	*10* ^−*2.5*^ *– 1*	**10** ^−**2**^, 10^−1.5^
*G*_*y*_	speed of adaptation predator	*10* ^−*2.5*^ *– 1*	**10** ^−**2**^

Marked in bold are the standard parameter values; marked in italics are parameters varied in small increments within sets of numerical simulations. Not all parameter combinations yielded sets that contained both ¼-lag and antiphase cycles; sets not containing both cycle types were excluded from the final analysis.

**Figure 1:**
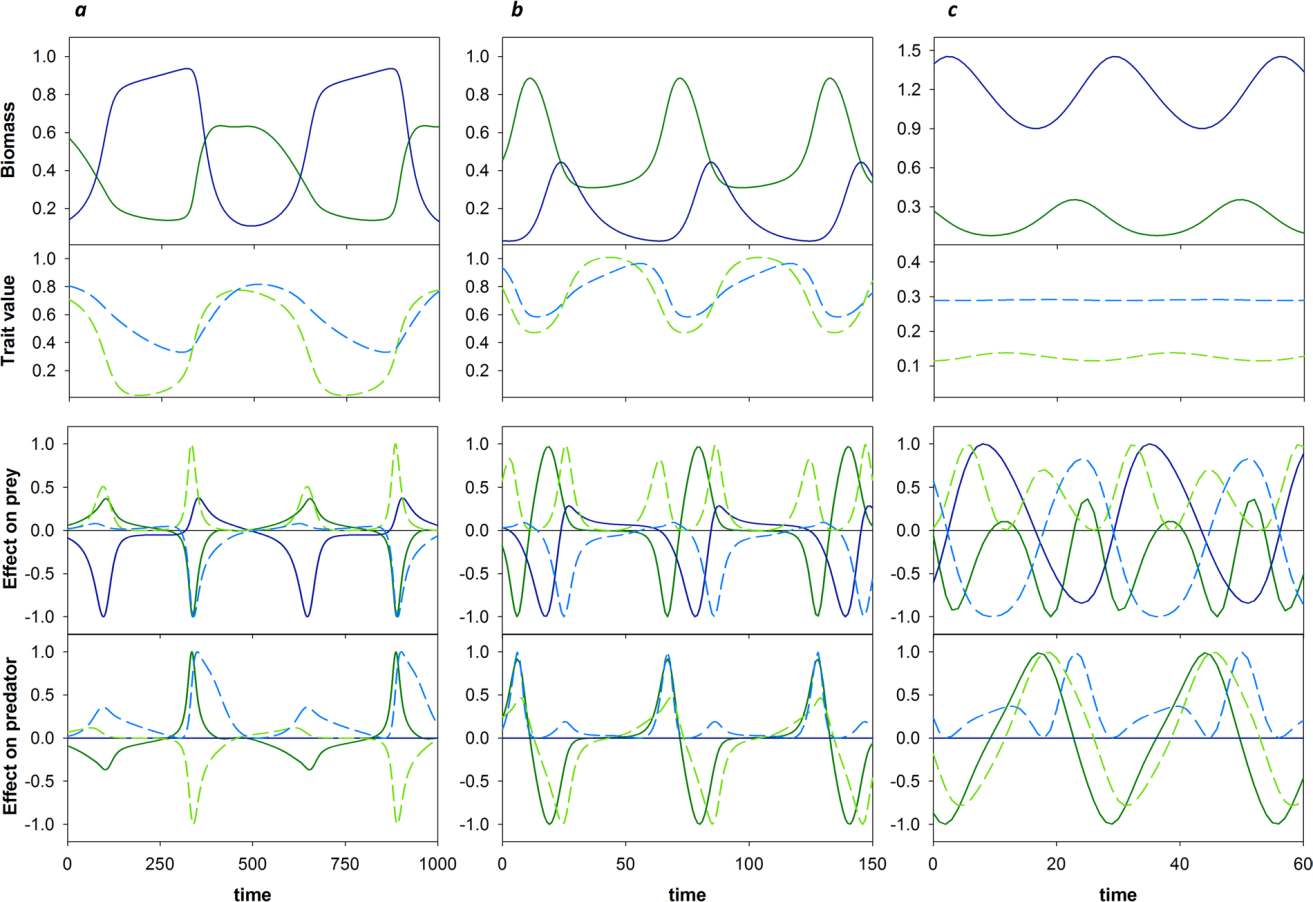
Eco-evolutionary dynamics and dynamics of components resulting from Geber method decomposition. Top row: prey (solid green) and predator (solid blue) dynamics. Second row: defense (dashed green) and offense (dashed blue) dynamics. Third row: how the change in the prey growth rate (*W*_*x*_) is affected by changes in prey biomass (*E*_*x*_^(*x*)^, solid green), predator biomass (*E*_*y*_^(*x*)^, solid blue), defense (*E*_*u*_^(*x*)^, dashed green) and offense (*E*_*v*_^(*x*)^, dashed blue). Bottom row: how the change in the predator growth rate (*W*_*y*_) is affected by the changes in prey biomass (*E*_*x*_^(*y*)^), predator biomass (*E*_*y*_^(*y*)^), defense (*E*_*u*_^(*y*)^) and offense (*E*_*v*_^(*y*)^). (**a**) Example of antiphase cycles, standard parameters for speed analysis (see Table 1) with *G*_*x*_ = *G*_*y*_ = 10^−2^. (**b**) ¼-lag cycles caused by rapid predator adaptation, standard parameters for speed analysis with *G*_*x*_ 10^−1^, *G*_*y*_ = 10^−0.9^. (**c**) ¼-lag cycles caused by high cost of defense, standard parameters for cost analysis with *c*_*x*_ = 5, *h* = 2. Time is measured in time steps after the first 30,000 time steps of the simulation. Component effects are standardized with respect to their absolute maxima over time, so the range is between −1 and 1.

Simulations in the speed analysis can result in either antiphase or ¼-lag predator-prey cycles, both characterized by distinct trait dynamics (Fig. 1a,b). In antiphase cycles, rapid high-amplitude changes in defense are followed by slower changes in offense, with especially the decrease in offense lagging behind that of defense (Fig. 1a). Conversely, in ¼-lag cycles the dynamics of offense track those of defense much more closely (Fig. 1b). The interaction between the traits results in other clear differences: the average levels of defense and offense are higher for ¼-lag cycles, and the amplitude of oscillations in both traits is smaller. Most strikingly, defense is maintained at a relatively high level even in the “undefended” phase of the cycle (Fig. 1b).

**Figure 2:**
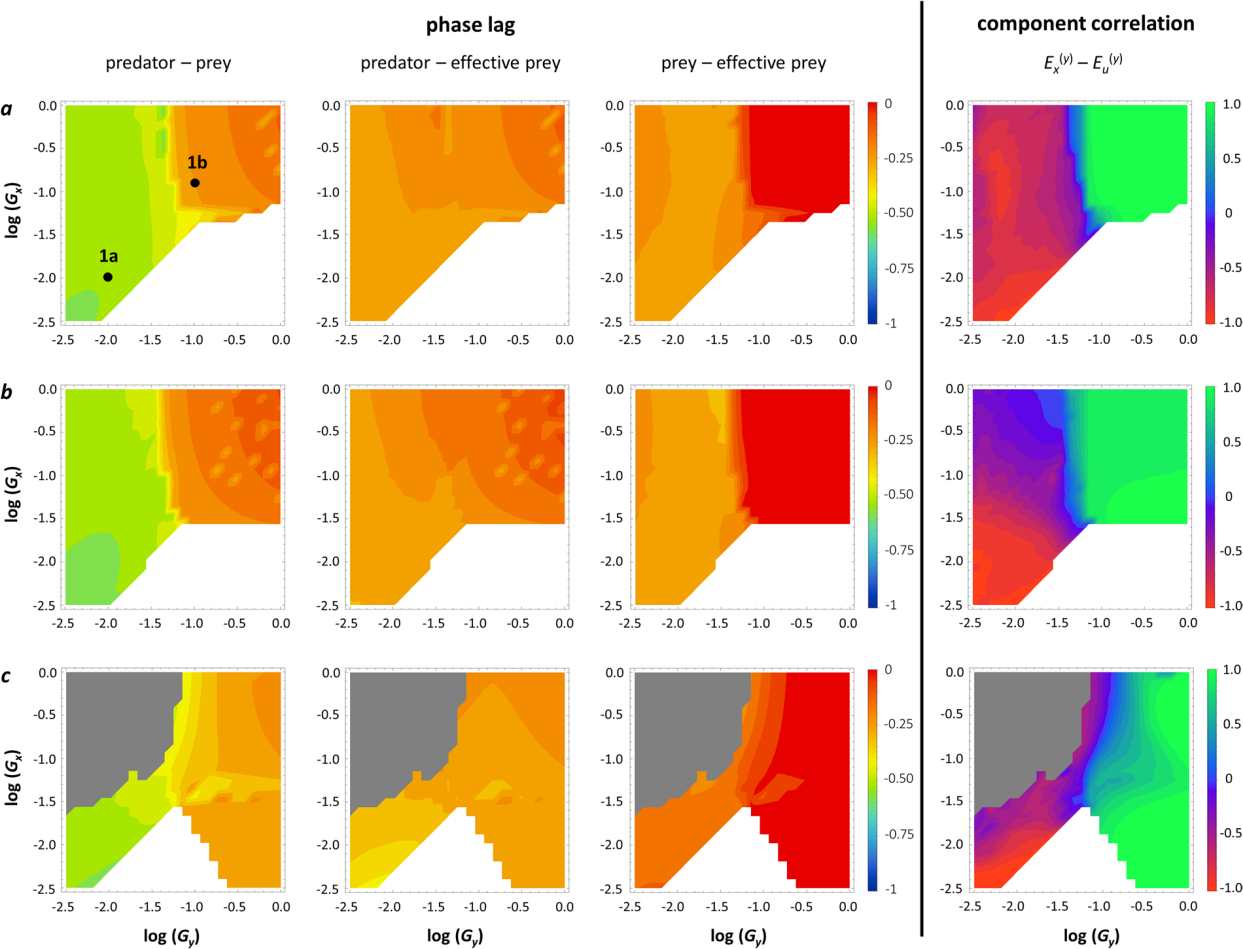
Effect of the speed of adaptation in prey and predator. Effect on phase relationships (left three columns) and the most significant component correlation (right), *E*_*x*_^(*y*)^ − *E*_*u*_^(*y*)^ (impact of prey biomass and defense on the net per capita predator growth rate; see Table 2). 1a and 1b refer to the parameter combinations for which the dynamics are shown in Fig. 1a and b, respectively. First column: phase lag between prey biomass and predator biomass; second column: phase lag between effective prey biomass and predator biomass; third column: phase lag between prey biomass and effective prey biomass. White: stable equilibrium; grey: extinction of predator. (**a**) standard parameters (see Table 1); (**b**) standard parameters except *K* = 2 (higher carrying capacity); (**c**) standard parameters except *h* = 2 (long handling time).

Both antiphase and ¼-lag cycles are consistently associated with parts of the parameter range studied: slow predator adaptation results in antiphase cycles, whereas rapid predator adaptation results in ¼-lag cycles. This pattern is highly consistent across all other parameters we varied (Fig. 2). Surprisingly, the speed of prey adaptation has little impact on the predator-prey phase relationship, although it may affect whether the predator persists (the predator may go extinct if prey adapt much more rapidly, especially if the handling time is long; Fig. 2c) or whether ongoing predator-prey cycles are found at all.

**Figure 3:**
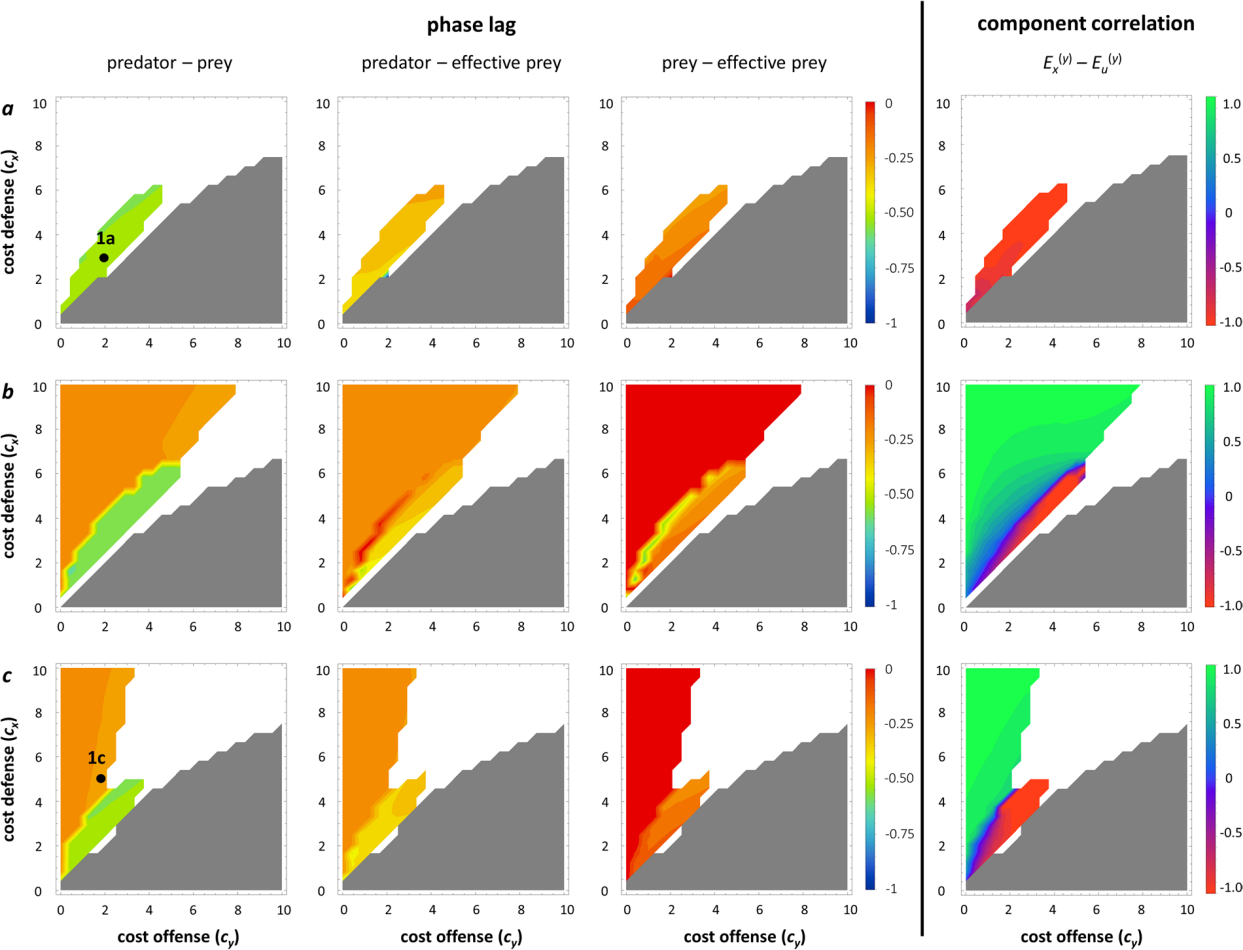
Effect of costs of defense and offense. Effect on phase relationships (left three columns) and the most significant component correlation (right), *E*_*x*_^(*y*)^ − *E*_*u*_^(*y*)^ (impact of prey biomass and defense on the net per capita predator growth rate; see Table 2). 1a and 1c refer to the parameter combinations for which the dynamics are shown in Fig. 1a and c, respectively. First column: phase lag between prey biomass and predator biomass; second column: phase lag between effective prey biomass and predator biomass; third column: phase lag between prey biomass and effective prey biomass. White: stable equilibrium; grey: extinction of predator. (**a**) Standard parameters for cost analysis; (**b**) standard parameters except *K* = 2 (high carrying capacity); (**c**) standard parameters except *h* = 2 (long handling time).

In the cost analysis, the relative costliness of defense and offense, rather than their absolute values, has the strongest impact on the nature of predator-prey dynamics including the phase relationship (Fig. 3). If offense is much more costly than defense, the predator invariably goes extinct, because it cannot achieve a sufficiently high attack rate to sustain itself. When both offense and defense are very costly, predator and prey stably coexist at equilibrium (Fig. 3a–c). A stable equilibrium may also be found when defense is costly while offense is cheap, but this requires that other parameters (mainly the prey carrying capacity *K* and handling time *h*) are relatively low, so that the ecological equilibrium would also be stable without adaptation (Fig. 3a). Antiphase cycles are here typically found when offense is slightly less costly than defense (Fig. 3). The eco-evolutionary dynamics in this parameter range are the same as in Fig. 1a. Conversely, ¼-lag cycles occur when defense is distinctly more costly than offense (Fig. 3b,c). It is worth noting that the eco-evolutionary dynamics of these ¼-lag cycles are very different from those caused by rapid predator adaptation (compare Fig. 1b,c): in this case, defense is limited to very low values due to the high costliness, and offense is always higher than defense. The amplitudes of ecological and especially evolutionary oscillations are small. Because the prey is always highly vulnerable to predation and the costs for the predator are relatively low, the predator maintains a high biomass while prey biomass is strongly depressed (Fig. 1c).

### Dynamics of effective prey biomass

**Figure 4:**
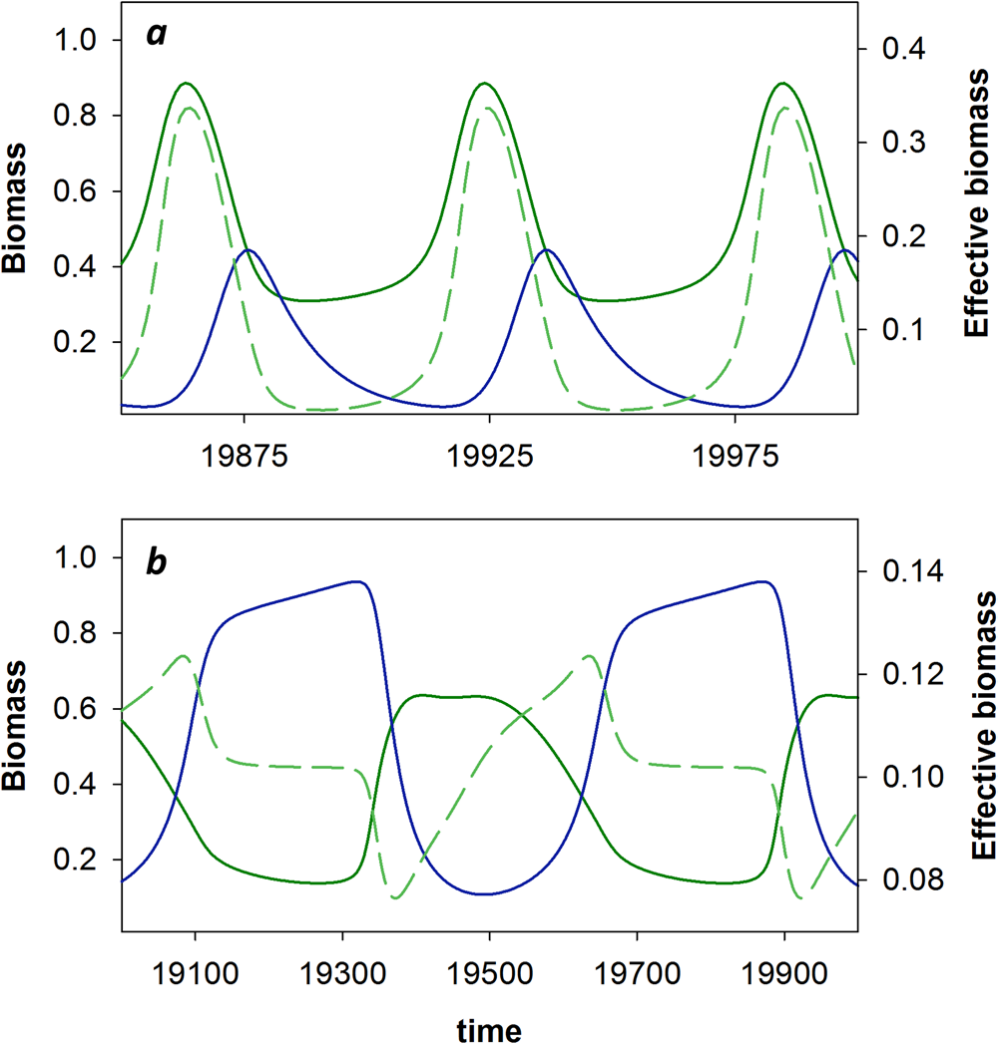
Dynamics of effective prey biomass. Prey (green) and predator (blue) biomass (solid lines) and effective prey biomass (dashed green lines). (**a**) Rapid predator adaptation resulting in ¼-lag cycles: *G*_*x*_ = *G*_*y*_ = 10^−1^. (**b**) Slow predator adaptation resulting in antiphase cycles: *G*_*x*_ = *G*_*y*_ = 10^−2^. Other parameters are standard for the speed analysis.

The predator peak always follows the peak in effective prey biomass, quantifying the amount of available prey biomass as perceived by the predator, with approximately a ¼-lag (Figs 2 and 3; second columns). Thus, the predator-prey phase relationship is generated by the phase relationship between the actual prey biomass and the effective prey biomass (Figs 2 and 3; third columns). A peak in effective prey biomass that coincides with the peak in actual prey biomass yields ¼-lag dynamics (Fig. 4a); however, if the peak in effective prey biomass is delayed with respect to the actual prey biomass, the predator-prey phase relationship is longer. If this delay is by a ¼-lag, predator and prey will be in antiphase (Fig. 4b).

### Component decomposition

To determine how prey and predator dynamics are affected by the four variables in the model (prey biomass *x*, predator biomass *y*, defense *u* and offense *v*), the Geber method^1^ decomposes the temporal dynamics of the prey and predator net per capita growth rates (*W*_*x*_ for prey, *W*_*y*_ for predators) into its four contributing components:

1 $$\begin{array}{c}\frac{d{W}_{x}}{dt}=\mathop{\underbrace{{E}_{x}^{(x)}}}\limits_{{\rm{Effect}}\,{\rm{of}}\,{\rm{prey}}}+\mathop{\underbrace{{E}_{y}^{(x)}}}\limits_{{\rm{Effect}}\,{\rm{of}}\,{\rm{predator}}}+\mathop{\underbrace{{E}_{u}^{(x)}}}\limits_{{\rm{Effect}}\,{\rm{of}}\,{\rm{defense}}}+\mathop{\underbrace{{E}_{v}^{(x)}}}\limits_{{\rm{Effect}}\,{\rm{of}}\,{\rm{offense}}}\\ \frac{d{W}_{y}}{dt}=\mathop{\underbrace{{E}_{x}^{(y)}}}\limits_{{\rm{Effect}}\,{\rm{of}}\,{\rm{prey}}}+\mathop{\underbrace{{E}_{y}^{(y)}}}\limits_{{\rm{Effect}}\,{\rm{of}}\,{\rm{predator}}}+\mathop{\underbrace{{E}_{u}^{(y)}}}\limits_{{\rm{Effect}}\,{\rm{of}}\,{\rm{defense}}}+\mathop{\underbrace{{E}_{v}^{(y)}}}\limits_{{\rm{Effect}}\,{\rm{of}}\,{\rm{offense}}}\end{array}$$

*E*_*x*_, *E*_*y*_, *E*_*u*_ and *E*_*v*_ are used throughout to denote the four factors influencing prey and predator net growth, with the superscripts (*x*) and (*y*) indicating whether they impact the prey or predator. For the full mathematical decomposition, see Methods.

### Component dynamics

The temporal dynamics of the eight components show a close relationship to the ecological and evolutionary dynamics (Fig. 1, third and bottom row), and are generally straightforward to interpret. First, an increase in predator biomass or in offense always has a negative impact on the prey, and a decrease in predator biomass or offense has a positive impact (see eq. (8) in the Methods section); thus, the components *E*_*y*_^(*x*)^ (Fig. 1, third row, solid blue line) and *E*_*v*_^(*x*)^ (Fig. 1, third row, dashed blue line) are positive when predator biomass and offense are decreasing, respectively, and negative when they are increasing. Similarly, an increase in prey biomass always has a positive impact on the predator, and an increase in defense always has a negative impact; thus, *E*_*x*_^(*y*)^ is positive when prey biomass is increasing and negative when prey biomass is decreasing, and the opposite is true for *E*_*u*_^(*y*)^ (Fig. 1, bottom row). Further, an increase in prey biomass generally has a negative impact on its own growth rate due to increased competition, causing *E*_*x*_^(*x*)^ to be negative when prey biomass is increasing, and positive when prey biomass is decreasing (Fig. 1, third row). The impact of predator biomass on its own growth, *E*_*y*_^(*y*)^, is always zero, since predator fitness does not depend on predator biomass (see Methods, eq. (5); Fig. 1, bottom row). Finally, the impact of defense on the prey *E*_*u*_^(*x*)^ is always positive, whether defense is increasing or decreasing, and the same is true for the impact of offense on the predator *E*_*v*_^(*y*)^ (Fig. 1, third and bottom row). This must by definition be true, as the dynamics of both traits follow the fitness gradient, and thus change in the direction that increases fitness. A detailed step-by-step description of the component dynamics, and how they relate to the eco-evolutionary dynamics, is given in Appendix C.

### Component correlation analysis

The component analysis reveals the mechanism that underlies the phase relationship between actual and effective prey biomass, and hence the predator-prey phase relationship. One component correlation is consistently associated with the predator-prey phase relationship in all analyses: the correlation between *E*_*x*_^(*y*)^ and *E*_*u*_^(*y*)^, that is, the impact of prey biomass and of defense on predator dynamics (Figs 2 and 3; Table 2). Several other component correlations are partly associated with the phase relationship, but none are consistent across analyses and they have little explanatory power (details in Appendix D).

**Table 2:** Component correlations and their association with the predator-prey phase relationship.

Component correlations (*r*_*C*_)						Association (*r*_*A*_ mean ± s.d.)	
Components		Speed analysis		Cost analysis			
Effect on	Effects of	antiphase	¼ lag	antiphase	¼ lag	Speed	Cost
Prey (*W*_*x*_)	*E*_*x*_^(*x*)^ − *E*_*u*_^(*x*)^	−/0	−/0	0	−	−0.17 ± 0.34	−0.24 ± 0.30
	*E*_*x*_^(*x*)^ − *E*_*v*_^(*x*)^	**+**	−	+	−/0/+	−**0.82 ± 0.08**	0.01 ± 0.32
	*E*_*y*_^(*x*)^ − *E*_*u*_^(*x*)^	−/0/+	−/0/+	−/**0**	**0/+**	−0.25 ± 0.42	**0.75 ± 0.08**
	*E*_*y*_^(*x*)^ − *E*_*v*_^(*x*)^	−	−	−	−	0.27 ± 0.33	0.07 ± 0.46
	*E*_*u*_^(*x*)^ − *E*_*v*_^(*x*)^	−/0	−/0	−/**0**	−	0.14 ± 0.49	−**0.77 ± 0.14**
Predator (*W*_*y*_)	*E*_*x*_^(*y*)^ − *E*_*u*_^(*y*)^	−	**+**	−	**+**	**0.91 ± 0.05**	**0.70 ± 0.14**
	*E*_*x*_^(*y*)^ − *E*_*v*_^(*y*)^	0	0	0	0	−0.05 ± 0.39	0.08 ± 0.34
	*E*_*u*_^(*y*)^ − *E*_*v*_^(*y*)^	−/0	0	−/0	0/+	0.58 ± 0.23	0.36 ± 0.30

Left columns: component correlations *r*_*C*_ found in the case of antiphase or ¼-lag cycles, categorized as positively correlated (*r*_*C*_ > 0), negatively correlated (*r*_*C*_ < 0) or uncorrelated (*r*_*C*_ ≈ 0). Multiple classifications are possible for each component correlation, as some component correlations are not consistent across all parameter values. Results in these columns are based on visual inspection; they are not used in the calculation of the associations (*r*_*A*_, see Methods). Right columns: associations *r*_*A*_ between the component correlations and the phase relationship.

In the case of antiphase cycles, the *E*_*x*_^(*y*)^ − *E*_*u*_^(*y*)^ component correlation is always strongly negative: the changes in the prey biomass and in defense always affect the predator in opposite directions, meaning that an increase in prey biomass always coincides with an increase in defense (Fig. 1a). As prey become more abundant, they simultaneously become less vulnerable to predation, causing the effective prey biomass to decline even though the actual prey biomass increases (Fig. 4b). Prey only become available to the predator when the latter has increased its level of offense, resulting in a pronounced delay in the peak in effective prey biomass (Fig. 4b), causing antiphase cycles between predator and prey biomass. Conversely, ¼-lag cycles occur when an increase in prey biomass coincides with a decrease in defense (Fig. 1b,c), resulting in a strong positive correlation between *E*_*x*_^(*y*)^ and *E*_*u*_^(*y*)^. Prey become more vulnerable to predation as they become more abundant, so that the attack rate is highest when prey are most abundant. This results in a peak in effective prey biomass coinciding with the peak in actual prey biomass (Fig. 4a), and predator-prey dynamics following a classic ¼-lag (Figs 2 and 3).

Thus, whether ¼-lag or antiphase cycles are found depends on whether prey biomass and defense cycle synchronously or anti-synchronously. Finally, the remaining question is what causes prey biomass and defense to cycle either synchronously or anti-synchronously. To explain this, we look again at the component dynamics (Fig. 1, third row). Because defense dynamics follow the direction of the fitness gradient, both increasing and decreasing defense impact the prey net per capita growth rate *W*_*x*_ positively, but these two effects are not necessarily of the same magnitude. Comparing the magnitudes of these two peaks within each timeseries shows the difference between antiphase and ¼-lag dynamics (see also Appendix C for a detailed view). When predator adaptation is slow, the positive impact of increasing defense is very pronounced (Fig. 1a, third row, dashed green line) as the shift from very low to high defense results in a strong release from predation. This strong positive impact on *W*_*x*_ causes a strong increase in prey growth, resulting in the prey biomass peak. The positive impact of decreasing defense on *W*_*x*_ is smaller, and cannot outweigh the negative impact of increasing predation (Fig. 1a, third row). The increase in defense thus directly causes the increase in prey biomass, explaining why they coincide.

In contrast, when the predator adapts rapidly, the positive impact of an increase in defense is limited by multiple factors (Fig. 1b): because defense never declines to very low values, the increase is less pronounced (going from semi-defended to highly defended), yielding lower benefit at relatively high costs. At the same time, the rapid evolutionary response of the predator largely cancels out the positive impact of increasing defense, limiting its positive impact even further. Instead, a release from the costs of defense generates the strongest positive net impact on prey growth under rapid predator evolution, causing the increase in prey biomass to coincide with decreasing defense. The same explanation holds for the ¼-lag dynamics caused by a high costliness of defense (Fig. 1c): the increases in defense are small, causing only minor reduction in predation, while the costs are high. For a detailed description of the transition from antiphase to ¼-lag cycles, see Appendix E.

## Discussion

Previous theory on the effects of coevolution^18–21^ leads to ambiguous predictions on how and why coevolution should impact predator-prey phase dynamics. To a large extent this is because limited effort has been spent in understanding the dynamics of complex eco-evolutionary models. Here we developed a new approach for understanding, on a fundamental level, the mechanisms underlying predator-prey phase dynamics. Applying this approach to a co-evolutionary predator-prey model, we could extract the mechanism underlying different phase relationships in predator-prey cycles. This mechanism rests on two results: (1) predator dynamics appear to follow the dynamics of effective prey biomass with a ¼-lag; and (2) the dynamics of effective prey biomass are generated in a highly predictable way by the relative importance of costs and benefits of prey defense. These general results are independent of the details of the model structure (see Appendix B).

In purely ecological models (i.e. without trait changes in either prey or predator), prey biomass and effective prey biomass are identical, and such models predict ¼-lag dynamics between predator and prey biomass^15^. For predator-prey cycles with a longer phase lag, including antiphase cycles, the dynamics of effective prey biomass must be delayed with respect to the actual prey biomass. This means that as prey become more abundant, either the attack rate, or the conversion efficiency of prey into predator biomass, or both must decrease. This is well captured by our main result: antiphase cycles occur when prey biomass and prey defense cycle synchronously, so peaks in prey biomass coincide with peaks in defense. When prey biomass and defense are anti-synchronized, the dynamics show classic ¼-lag cycles.

Our analysis further gives a mechanistic explanation for this pattern. For prey biomass and defense to become synchronized, an increase in defense must have a strong positive net impact on the prey net growth rate: the resulting strong release from predation causes the increase in prey biomass. This is expected to be the case when the effectiveness of defense is high, especially when this is combined with low costliness. Conversely, prey biomass and defense become anti-synchronized when it is not the increase, but the decrease in defense that has the strongest net positive impact on the net prey growth rate. For this to happen, the benefits of increasing defense should be limited (e.g. due to a rapidly-adapting predator) compared to the costs. In a very short summary, antiphase cycles occur when the peak in prey biomass is caused by a release from predation (top-down effects outweigh bottom-up effects), whereas ¼-lag cycles occur when the peak in prey biomass is caused by a release from the costs of defense (bottom-up effects outweigh top-down effects). Both scenarios are realistic: natural phytoplankton blooms can consist of undefended or of highly defended prey^24^.

All predictions of our model are consistent with previous experimental and theoretical work on how rapid evolution affects predator-prey cycles. Antiphase cycles occurred in both experiments and theory when only prey can evolve^10,14,16,17,25^, which may be considered the extreme limit of slow predator adaptation. Studies using two distinct prey clones revealed that antiphase cycles only occur when defense is effective, i.e. when the defended clone has a very low palatability^12,16^. Our strongest prediction, that in antiphase cycles the effects of evolutionary and ecological changes in the prey should affect the predator in opposite directions, is experimentally confirmed^14^. Further, we predict that antiphase cycles will show prey peaks consisting of highly defended prey, and/or a simultaneous increase in prey biomass and defense. This is consistent with dynamics shown in previous studies on prey evolution alone^12,17,26^ and theory on predator-prey coevolution^18–20^.

We find that the necessary requirements for antiphase cycles are twofold: (1) the increase in defense must result in a strong rapid release from predation; and (2) there must be an appreciable delay in the predator’s evolved countermeasures. In our model, these conditions are met when predator adaptation is slow, regardless of the speed of adaptation in the prey, which agrees with a previous modelling study using a very similar model structure^18^. However, in other models or real systems the above conditions may be met in a different way, revealing why predictions of previous modelling studies have been contradictory. For example, depending on details of model structure, slow predator adaptation in a strict sense may be neither a necessary nor a sufficient condition. Antiphase cycles were found in models studying the fast evolution limit, in which evolutionary change is instantaneous compared to ecological dynamics^21^, generating sudden shifts between extreme phenotypes (undefended and highly defended prey, non-offensive and highly offensive predators). However, both conditions are still met, as the predator’s evolutionary response always followed the shift in defense at a distinct delay. Similarly, a striking recent experimental and modelling study on host-virus coevolution^9^, with potentially very rapid predator (i.e. viral) evolution, shows antiphase cycles with exactly the pattern our model predicts: the emergence of a resistant mutant in a susceptible host population results in a rapid simultaneous increase in defense and in host density. Here the effectiveness of each defense mutation is very high (going from highly susceptible to the current virus population to highly resistant), and predator adaptation is delayed because a de novo mutation is necessary to counteract defense, thus meeting both criteria predicted by our analysis.

Predator evolution has often been neglected in studies on eco-evolutionary dynamics. Our results show that the speed of predator adaptation may indeed be more decisive in determining the nature of predator-prey dynamics than the speed of prey adaptation. Even more strikingly, we suggest that the dynamics caused by rapid adaptation in both predator and prey may look indistinguishable from predator-prey dynamics with no adaptation at all. This implies the possibility that antiphase cycles being a “smoking gun” for rapid adaptation may partly be an artefact caused by such studies typically only including prey adaptation. Furthermore, the effect of rapid predator evolution in our model is mostly driven by a feedback between defense and offense dynamics. This illustrates the importance of studying systems in which such real-time feedbacks are possible, such as the model presented here, as these feedbacks may impact eco-evolutionary dynamics in ways that cannot be captured by assuming separate timescales^17,20,21^. At the same time, it also urges caution against extrapolating too freely from model results, as such feedbacks and their impact on predator-prey dynamics may be model- or system-specific. Focusing on understanding the mechanisms underlying it, however, allows us both to explain contradictions in previous models and to make general predictions.

Across all our simulations, the dynamics we found were either the classic ¼-lag cycles also found in ecological models^15^ or the “classic” eco-evolutionary antiphase cycles^10,12,16^. At the transition between these two dynamics, intermediate phase lags may occur over a narrow parameter range (see Appendix E for a detailed description and explanation); apart from this, we did not observe other deviations from the classic ¼-lag such as reversed cycles^20^. This is most likely due to differences in model assumptions: reversed cycles have been shown to require disruptive selection on prey and predator traits, resulting in rapid shifts between extreme trait values^20^, while we model gradual trait changes. Whether reversed cycles or other deviations from the ¼-lag are expected or even possible in the type of model we use here is a subject for future study.

Here we studied a model with a specific structure, assuming a unidirectional trait axis to explain the distinct feedbacks leading to antiphase and ¼-lag cycles. However, our approach is very general, and can be applied to systems with bidirectional evolution^19^ or reciprocal phenotypic plasticity^22,27,28^, and to answer different questions. For example, phenotypic plasticity has been indicated to result in very different phase dynamics from evolution^25^, although this appears to depend on the mechanism regulating plasticity^22^. Our approach can elucidate what causes the differences between these different scenarios, with the potential to reach a broad overarching understanding of how trait and biomass dynamics interact to generate eco-evolutionary dynamics.

## Methods

### Model structure

Following previous work on coevolution^18–21,29^, we model a single prey (*x*) and predator (*y*), each with an adaptive trait (defense *u* and offense *v*, respectively); the two traits together determine the capture rate of the predator (see Appendix A, Fig. A1a,b). The ecological predator-prey dynamics are described as follows:

2 $$\begin{array}{c}\frac{dx}{dt}=(r(u)(1-\frac{x}{K})-\frac{a(u,v)y}{1+a(u,v)hx})x\\ \frac{dy}{dt}=(g(v)\frac{a(u,v)x}{1+a(u,v)hx}-d)y\end{array}$$

Prey grow logistically, where *r*(*u*) and *K* are the intrinsic growth rate and carrying capacity. Predation follows a Holling type II functional response, with attack rate *a*(*u*, *v*) and handling time *h*. Finally, *g*(*v*) is the conversion efficiency of captured prey into predator biomass, and *d* is the per capita predator mortality rate. The levels of defense and offense together determine the predator attack rate *a*(*u, v*). We assume that predation always decreases with increasing *u* (defense) and increases with increasing *v* (offense):

3 $$a(u,v)=\frac{{a}_{0}}{1+{e}^{\theta (u-v)}}$$

*a*_0_ is the maximum attack rate, achieved if *v* ≫ *u* (high offense and low defense). Conversely, if defense is very high compared to offense (*u* ≫ *v*), the attack rate approaches zero. *θ* determines the steepness of the transition between high and low attack rates (Appendix A, Fig. A1b). This represents what has been called a unidirectional trait axis^13^, a common assumption in models of coevolution that is applicable to many predator-prey interactions^30^.

We assume that defense trades off with the prey intrinsic growth rate *r* and that offense trades off with the predator conversion efficiency *g*:

4 $$\begin{array}{c}r(u)={r}_{0}{e}^{-{c}_{x}{u}^{2}}\\ g(v)={g}_{0}{e}^{-{c}_{y}{v}^{2}}\end{array}$$

*r*_0_ and *g*_0_ are the maximum growth rate and conversion efficiency, attained when *u* = 0 and *v* = 0, respectively. *c*_*x*_ and *c*_*y*_ determine how rapidly the realized growth rate *r*(*u*) and conversion efficiency *g*(*v*) decrease with increasing defense and offense; thus, they denote the costliness of defense and offense (Appendix A, Fig. A1c-d). This trade-off shape was chosen because it is also valid for a bidirectional trait axis^19^, making direct comparison between these two scenarios in a future study possible. To ascertain the robustness of our results with respect to the specific trade-off functions, we also analyzed the model with a different trade-off, which gave rise to highly similar results and supported the generality of our conclusions (Appendix B).

The evolutionary dynamics of *u* and *v* are modelled using the quantitative genetics approach^31^. The speed and direction of evolutionary change is proportional to the fitness gradient, evaluated at the current trait value. Prey and predator fitness are defined as the per capita net growth rates (i.e. Malthusian fitness) *W*_*x*_ and *W*_*y*_:

5 $$\begin{array}{c}{W}_{x}=\frac{1}{x}\frac{dx}{dt}=r(u)(1-\frac{x}{K})-\frac{a(u,v)y}{1+a(u,v)hx}\\ {W}_{y}=\frac{1}{y}\frac{dy}{dt}=g(v)\frac{a(u,v)x}{1+a(u,v)hx}-d\end{array}$$

Evolutionary dynamics of the two traits are then described by

6 $$\begin{array}{c}\frac{du}{dt}={G}_{x}\frac{\partial {W}_{x}}{\partial u}{e}^{-\varepsilon /u}\\ \frac{dv}{dt}={G}_{y}\frac{\partial {W}_{y}}{\partial v}{e}^{-\varepsilon /v}\end{array}$$

*G*_*x*_ and *G*_*y*_ represent the additive genetic variation in the prey and predator populations, determining the speed of evolutionary change relative to the ecological dynamics (*G*_*x*_ = *G*_*y*_ = 1 would indicate ecological and evolutionary dynamics are equally fast). In line with previous modelling studies^18–20,22^, we keep *G*_*x*_ and *G*_*y*_ constant within each simulation run. The exponential functions in eq. (6) are boundary functions restricting the dynamics of *u* and *v* to positive values by decreasing the speed of evolutionary change when *u* or *v* very closely approach zero (*ε* = 0.001).

### Effective prey biomass

In addition to the biomasses and trait values (*x*, *y*, *u*, *v*), we consider the dynamics of the effective prey biomass: the prey biomass as it is perceived by the predator. We define the effective prey biomass here as a combination of three elements:

1. Prey biomass *x*; we refer to this as “actual prey biomass” throughout the manuscript when contrasting it with the effective prey biomass.
2. The vulnerability of the prey to the predator. This is influenced by any form of defense allowing the prey to avoid being captured by the predator: examples include formation of colonies too large for predators to ingest^12^ or resistance against viral infections^9^ It is also influenced by the predator’s countermeasures against defense, such as increases in gape size to capture larger prey^27^ or increased virulence^9,32^.
3. The fraction of captured prey biomass that can actually be digested and converted into predator biomass. This may be lowered by forms of defense inhibiting digestion; for example, differences in size or cell wall structure may make it more likely for defended algae to survive gut passage^33,34^. Less intuitively, this third element is also affected by predators expending more energy in high-offense strategies, as the energetic costs of offense lowers the conversion efficiency of captured prey into predator biomass^35,36^.

Reflecting the above, in the case of our model we calculate the effective prey biomass by multiplying the prey biomass *x* with the predator’s attack rate *a* relative to the maximum attack rate *a*_0_, and the predator’s conversion efficiency *g* relative to the maximum conversion efficiency *g*_0_:

7 $${x}_{eff}=x\cdot \frac{a(u,v)}{{a}_{0}}\cdot \frac{g(v)}{{g}_{0}}$$

It should be noted that the definition of the effective prey biomass may be different depending on the details of the system under study. We assume here that the attack rate is a function of both traits, whereas the conversion efficiency is only a function of the level of offense. If defense takes a form where it allows prey to escape digestion rather than, or in addition to, escaping capture, the conversion efficiency should be expressed as a function of the prey trait as well. Additionally, defense can take the form of toxic compounds negatively affecting predator survival^37^; similarly, high levels of offense in the predator may come at the cost of increased mortality rather than lowering the conversion efficiency^29,36^. Such scenarios may call for a different definition of the effective prey biomass, but this does not affect the principal concept or our results and conclusions (see Appendix B).

### Phase relationships

The phase lag *φ* between predator and prey was calculated using the dominant frequency of the Fourier transform of the last 20,000 time steps of the simulated time series comprising 50,000 time steps. It is expressed as −1 ≤ *φ* ≤ 0, where 0 and −1 indicate in phase cycles (no lag), *φ* = −0.5 indicates antiphase cycles, and *φ* = −0.25 classic ¼-lag cycles.

### Geber method decomposition and component correlations

The change in per capita net prey growth (fitness, *W*_*x*_; see eq. (5)) is the result of how prey fitness is impacted by changes in prey biomass (*x*), predator biomass (*y*), defense (*u*) and offense (*v*); the same holds true for the predator fitness (*W*_*y*_). Using this principle, with the Geber method^1^ we decompose the temporal dynamics of *W*_*x*_ and *W*_*y*_ into their four variable components:

8 $$\begin{array}{c}\frac{d{W}_{x}}{dt}=\mathop{\underbrace{\frac{\partial {W}_{x}}{\partial x}\frac{dx}{dt}}}\limits_{{\rm{Effect}}\,{\rm{of}}\,{\rm{prey}}\,({E}_{x}^{(x)})}+\mathop{\underbrace{\frac{\partial {W}_{x}}{\partial y}\frac{dy}{dt}}}\limits_{{\rm{Effect}}\,{\rm{of}}\,{\rm{predator}}\,({E}_{y}^{(x)})}+\mathop{\underbrace{\frac{\partial {W}_{x}}{\partial u}\frac{du}{dt}}}\limits_{{\rm{Effect}}\,{\rm{of}}\,{\rm{defense}}\,({E}_{u}^{(x)})}+\mathop{\underbrace{\frac{\partial {W}_{x}}{\partial v}\frac{dv}{dt}}}\limits_{{\rm{Effect}}\,{\rm{of}}\,{\rm{offense}}\,({E}_{v}^{(x)})}\\ \frac{d{W}_{y}}{dt}=\mathop{\underbrace{\frac{\partial {W}_{y}}{\partial x}\frac{dx}{dt}}}\limits_{{\rm{Effect}}\,{\rm{of}}\,{\rm{prey}}\,({E}_{x}^{(y)})}+\mathop{\underbrace{\frac{\partial {W}_{y}}{\partial y}\frac{dy}{dt}}}\limits_{{\rm{Effect}}\,{\rm{of}}\,{\rm{predator}}\,({E}_{y}^{(y)})}+\mathop{\underbrace{\frac{\partial {W}_{y}}{\partial u}\frac{du}{dt}}}\limits_{{\rm{Effect}}\,{\rm{of}}\,{\rm{defense}}\,({E}_{u}^{(y)})}+\mathop{\underbrace{\frac{\partial {W}_{y}}{\partial v}\frac{dv}{dt}}}\limits_{{\rm{Effect}}\,{\rm{of}}\,{\rm{offense}}\,({E}_{v}^{(y)})}\end{array}$$

Our goal was to determine the presence and strength of any positive or negative interactions between these components. A positive correlation between two components indicates that they generally affect the prey or predator in the same direction over time, thus reinforcing one another. For instance, an increase in prey biomass (positive impact on the predator) occurring simultaneously with a decrease in defense (also positive impact on the predator), and a decrease in prey biomass occurring simultaneously with an increase in defense, would yield a positive correlation between *E*_*x*_^(*y*)^ and *E*_*u*_^(*y*)^. Conversely, a negative correlation indicates that the two components generally act in opposition to each other. For a detailed description on how to interpret these component dynamics, see Appendix C.

To find such interactions, we calculated the component correlations *r*_*C*_ as the Spearman rank correlation coefficients between all component pairs over the last 5000 time steps of the simulations. As there are 4 components for *W*_*x*_ and 4 for *W*_*y*_, there are potentially 12 component correlations in total (6 for *W*_*x*_ and 6 for *W*_*y*_). However, we disregarded the component correlations *E*_*x*_^(*x*)^ − *E*_*y*_^(*x*)^ and *E*_*x*_^(*y*)^ − *E*_*y*_^(*y*)^, since these correlations are a reflection of the predator-prey phase relationship rather than explanatory factors. Furthermore, *E*_*y*_^(*y*)^ is always zero, so the *E*_*y*_^(*y*)^ − *E*_*u*_^(*y*)^ and *E*_*y*_^(*y*)^ − *E*_*v*_^(*y*)^ component correlations are not meaningful. Thus, a total of 8 component correlations were calculated per simulation (5 for *W*_*x*_ and 3 for *W*_*y*_).

### Numerical simulations and final association analysis

For the numerical simulations, we varied the focal parameters (*G*_*x*_ and *G*_*y*_ for the speed analysis, *c*_*x*_ and *c*_*y*_ for the cost analysis) on a fine grid of 25 × 25 combinations, keeping all other parameters constant. Each grid of 25 × 25 combinations constitutes one set. To generate a large number of such sets and to test the generality of our analysis, we varied other model parameters; see Table 1 for details.

Finally, our goal was to determine whether any component correlations were consistently different between antiphase and ¼-lag cycles, and may thus be a predictor for the phase relationship. For this, within each set we determined the predator-prey phase relationship *φ* and all component correlations for all 25 × 25 parameter combinations. Sets that contained only ¼-lag or only antiphase cycles were discarded from further analysis. We then calculated the rank correlations *r*_*A*_ (“associations”) between *φ* and each component correlation, yielding 8 associations per set. This resulted in a final data set of 8 × 82 associations (8 component correlations in 82 sets included in the final analysis; see Tables 1 and 2).

### Data availability

All data used in this study are available from the corresponding author on request.

## Electronic supplementary material

Supplementary Material
