# Supplementary material for: Disentangling eco-evolutionary dynamics of predator-prey coevolution: the case of antiphase cycles
Source: Sci Rep. 2017 Dec 7;7:17125. doi: 10.1038/s41598-017-17019-4 (PMC5719453; doi:10.1038/s41598-017-17019-4)
Supplement: Supplementary file 1 — Supplementary Material [file 41598_2017_17019_MOESM1_ESM.pdf]

Supplementary Information

Ellen van Velzen, Ursula Gaedke, *Disentangling eco-evolutionary dynamics of predator-prey coevolution: the case of antiphase cycles*

Appendix A: Model structure

We consider a model with a single prey and a single predator species or phenotype, each with an adaptive trait determining the vulnerability of the prey to predation. Both traits are subject to a trade-off: an increased investment in defense in the prey lowers its growth rate $r(u)$, and an increased investment in offense in the predator lowers its conversion efficiency $g(v)$ (Fig. A1a).

Attack rate

The vulnerability of the prey to predation is determined by the attack rate $a(u,v)$. We assume a unidirectional trait axis[1]: investing more in defense means the prey becomes less vulnerable to predation, regardless of the predator's current investment in offense. Likewise, predators investing more in offense have a higher prey capture rate, regardless of the level of defense in the prey. The attack rate $a(u,v)$ is thus a function of the difference between the two trait values (Fig. A1b). This trait structure is applicable to many types of defense-offense coevolution, such as arms races in swimming speed in prey vs. predators, toxin-antitoxin coevolution or weapon-armour coevolution[2-5].

Trade-offs for prey and predator traits

Both traits are assumed to come at a cost. A higher level of defense reduces the prey intrinsic growth rate $r(u)$, as has been shown to be the case in algae[6, 7]. A higher level of offense in the predator reduces the conversion efficiency of captured prey into predator biomass, as a higher proportion of captured resources are required to maintain a higher level of offense. For example, if offense is swimming speed in zooplankton, metabolic costs are associated with a high level of offense[8, 9]; the same should be true when investment into antitoxins or specific structures to capture prey. Little is known about the exact shape of such trade-offs. Like others[10, 11] we assume that prey growth rate r and conversion efficiency g reach their maxima at u, $v = 0$ (no investment) and converge to zero at very high trait values. Assuming a bell-shaped curve for both trade-offs[11] (Fig. A1c, A1d) allows us to compare this model with unidirectional traits to a model with a bidirectional trait axis, such as size-specific predation[1]. Reasonable trade-offs with different shapes or affecting different traits can be postulated; see Appendix D for an example.

Evolutionary dynamics

The evolutionary dynamics of u and v are modelled using the quantitative genetics approach[12]. The speed and direction of evolutionary change is proportional to the fitness gradient, evaluated at the current trait value. Prey and predator fitness are defined as the per

capita net growth rates (i.e. Malthusian fitness) W_x and W_y; evolutionary dynamics of the two traits are then described by

$$\begin{aligned}\frac{du}{dt} &= G_x \frac{\partial W_x}{\partial u} e^{-\varepsilon/u}\\ \frac{dv}{dt} &= G_y \frac{\partial W_y}{\partial v} e^{-\varepsilon/v}\end{aligned} \tag{A1}$$

where G_x and G_y represent the additive genetic variation in the prey and predator populations.

This approach assumes frequency-independent selection, where fitness of an individual is affected only by its own trait value, not by that of others in the population. With defense and offense traits as defined in our model, this is the case. For defense, neither the benefit (i.e. reduced attack rate) nor the costs (i.e. reduced intrinsic growth rate) are affected by defense strategies of conspecifics; the same is true for the benefits (increased attack rate) and costs (decreased conversion efficiency) of offense for the predator.

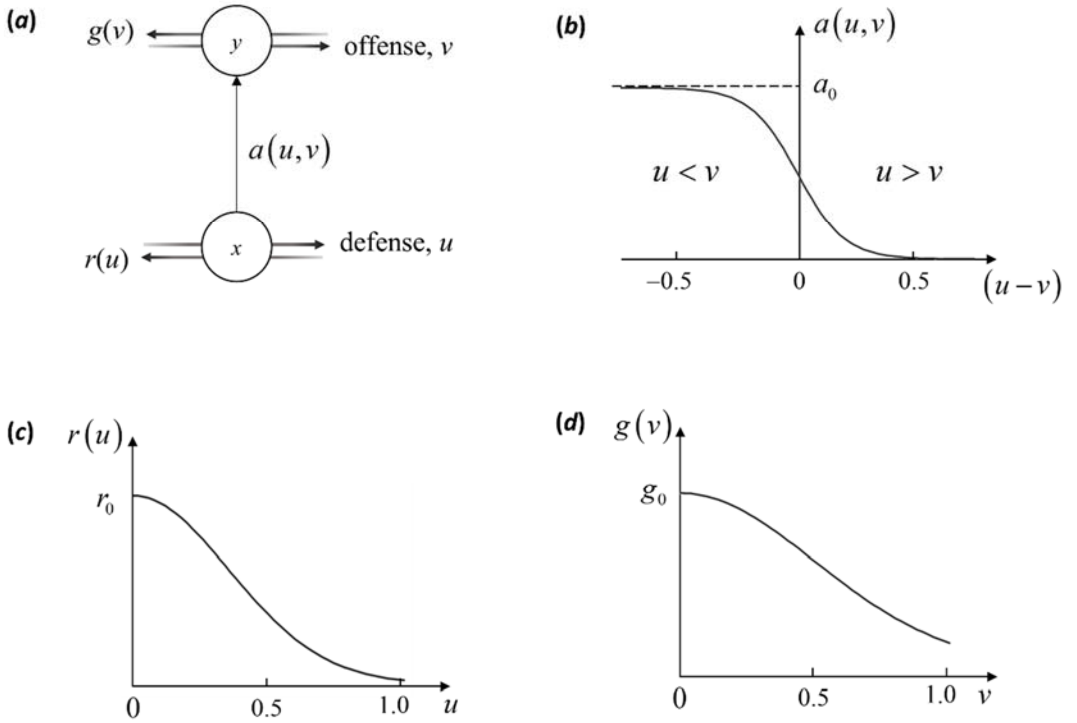

Figure A1: Model structure and trade-offs. (a) Basic structure of the model with four variables: x (prey biomass), y (predator biomass), u (defense) and v (offense). (b) Attack rate, depending on the difference between offense v and defense u, determining the vulnerability of the prey to predation, here shown for $\theta = 10$ (standard parameter value for all simulations). (c) Trade-off between defense u and prey intrinsic growth rate $r(u)$, here shown for $c_x = 3$ (standard value for speed analysis). (d) Trade-off between offense v and predator conversion efficiency $g(v)$, here shown for $c_y = 2$ (standard value for speed analysis). The standard value for c_x is higher than for c_y because there are no oscillations for $c_y \geq c_x$ (see Figure 3 in main text).

Appendix B: The impact of trade-off structure

To test the generality of our result, we analyzed the model with a different trade-off for the predator, where increasing offense comes at the cost of higher mortality, rather than a lower conversion efficiency. A mortality trade-off has been assumed by some previous models on mutual adaptation[11, 13]. This kind of trade-off may arise when higher investment in offense decreases the predator's defense against its own predators or increases the risk of starvation, or when higher predator activity levels (i.e. higher offense) increases the encounter rates with both its prey and with its predators[9]. Mortality is thus expressed as a function of offense *v*:

$$d(v) = d_0 + d_1 (1+v)^{c_y} \tag{B1}$$

Here, d_0 and d_1 together determine the basal mortality rate when investment into offense is minimal ($v(t) = 0$), and d_1 and c_y determine the shape of the trade-off, with higher values of c_y yielding a stronger trade-off, i.e. higher costs for offense. The values used for all parameters can be found in Table B1.

In this case, the conversion efficiency is not dependent on the offense trait; the effective prey biomass thus becomes a combination only of prey biomass and attack rate:

$$x_{eff} = x \cdot \frac{a(u,v)}{a_0} \tag{B2}$$

It can be argued that this calculation of the effective prey biomass is not entirely equivalent to that in the main model (see eq. (7)), as the costs of offense are here neglected entirely. Implementing these costs in a biologically meaningful way is not straightforward, and we make no attempt here; however, this is the reason why the phase relationship between prey biomass and effective prey biomass as calculated in eq. (B2) may deviate here from a strict ¼-lag (Fig. B1, B2).

Results

None of the results of this model deviate substantially from those of the original model. The results of the speed analysis are nearly indistinguishable (compare Fig. B1 with Fig. 2 in the main Results): slow predator adaptation results in antiphase cycles, regardless of the speed of adaptation in the prey, and rapid predator adaptation results in ¼-lag cycles. A minor difference is that a combination of very rapid prey adaptation and slow predator adaptation more easily drives the predator extinct (upper left corners in Fig. B1).

Similarly, the results of the cost analysis strongly resemble those of the original model. A high costliness for defense results in ¼-lag cycles, whereas a high costliness for offense results in the extinction of the predators. Intermediate values for both c_x and c_y generate antiphase cycles (Fig. B2). A lower baseline mortality for the predator (low d_0) results in antiphase cycles over a wider parameter range (Fig. B2b), because it increases the range for c_y where offense can keep up with defense.

In the association analysis, it is clear that the component correlation $E_x^{(y)}$ - $E_u^{(y)}$ is again the strongest predictor for the phase relationship (Table B2): as in the original analysis, ¼-lag

cycles are associated with a strong positive correlation between these two components, while antiphase cycles are associated with a strong negative correlation (Fig. B1, B2; Table B2).

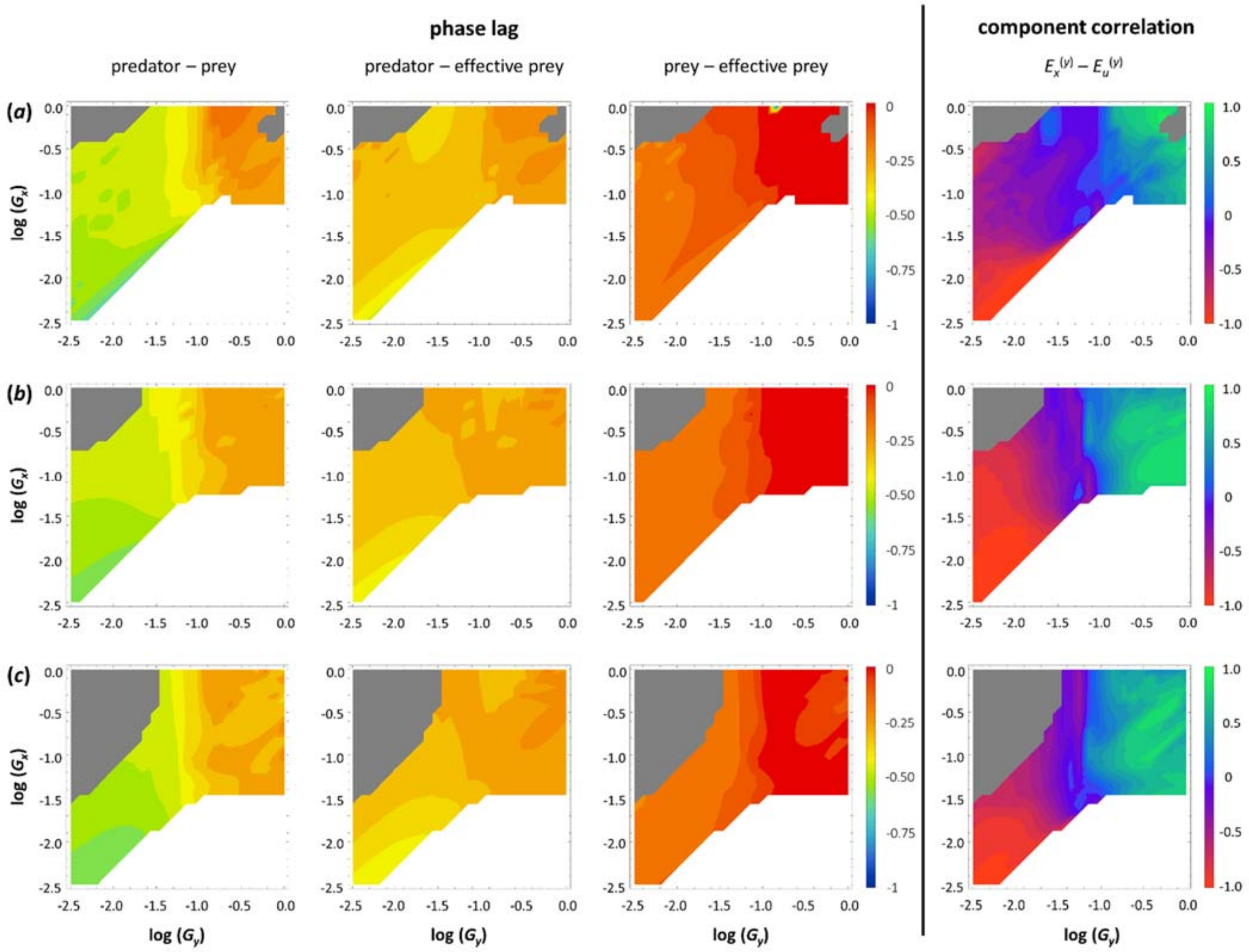

Figure B1: Effect of the speed of adaptation in prey and predator on phase relationships (left three columns) and the most significant component correlation (right), $E_x^{(y)}$ - $E_u^{(y)}$ (see Table B2) for the model with a mortality trade-off for the predator. First column: phase lag between prey biomass and predator biomass; second column: phase lag between effective prey biomass and predator biomass; third column: phase lag between prey biomass and effective prey biomass. White: stable equilibrium. Grey: extinction of predators. (a) standard parameters (see Table B1); (b) standard parameters except $c_x = 4.0$, $c_y = 3.0$ (higher costliness for both traits); (c) standard parameters except $c_x = 4.0$, $c_y = 3.0$ and $d_0 = 0.01$ (lower predator mortality constant).

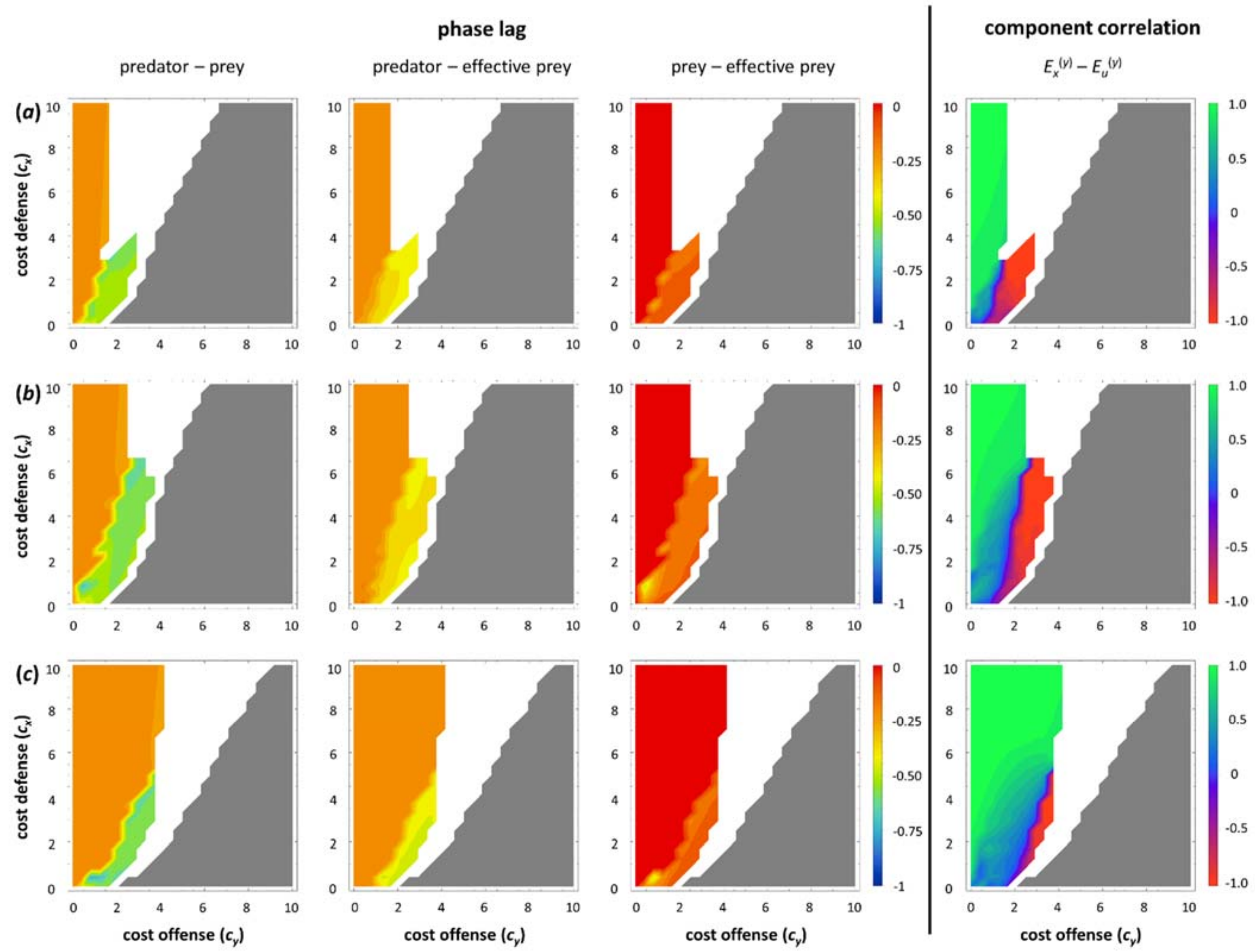

Figure B2: Effect of costliness of defense and offense on phase relationships (left three columns) and the most significant component correlation (right), $E_x^{(y)} - E_u^{(y)}$ (see Table B2) for the model with a mortality trade-off for the predator. First column: phase lag between prey biomass and predator biomass; second column: phase lag between effective prey biomass and predator biomass; third column: phase lag between prey biomass and effective prey biomass. White: stable equilibrium. Grey: extinction of predators. (a) standard parameters for cost analysis except $h = 2$ (long handling time); (b) standard parameters except $h = 2$ and $d_0 = 0.01$ (long handling time and low predator mortality constant); (c) standard parameters except $K = 2$ (high carrying capacity).

Appendix B

Table B1: Parameters and values used in the mortality trade-off version of the model. Standard parameter values are marked in bold; in italics are the parameters varied on a fine grid in the speed analysis (G_x, G_y) and cost analysis (c_x, c_y). Under standard parameters for d_0 and d_1, total mortality at v = 0 (no investment in offense) is the same as in the original model formulation (d = 0.1; see Table 1 in main text).

Parameter	Description	Value	
		Speed analysis	Cost analysis
K	carrying capacity	**1.0**, 1.5	**1.0**, 1.5, 2.0
r_0	maximum growth rate prey	**1.0**	**1.0**
d_0	mortality constant predator	0.01, **0.05**	0.01, **0.05**
d_1	basal mortality predator	**0.05**	**0.05**
g	conversion efficiency	**1.0**	**1.0**
θ	efficiency of defense	**10**	**10**
a_0	maximum attack rate	**1.0**	0.5, 0.75, **1.0**, 1.25, 1.5
h	handling time predator	**1.0**	**1.0**, 1.5, 2.0
c_x	costliness of defense	2.0, **3.0**, 4.0, 5.0	*0 – 10*
c_y	costliness of offense	**2.0**, 3.0, 4.0, 5.0	*0 – 10*
G_x	speed of adaptation prey	*$10^{-2.5}$ – 1*	**10^{-2}**
G_y	speed of adaptation predator	*$10^{-2.5}$ – 1*	**10^{-2}**

Table B2: Component correlations and their association with the predator-prey phase relationship. Left columns: component correlations r_C found in the case of antiphase or ¼-lag cycles, categorized as positively correlated ($r_C > 0$), negatively correlated ($r_C < 0$) or uncorrelated ($r_C \approx 0$). Results in this column are based on visual inspection; they are not used in the calculation of the associations (r_A, see Methods). Right columns: associations r_A between the component correlations and the phase relationship.

Component correlations (r_C)						Association (r_A mean ± s.d.)	
Components		Speed analysis		Cost analysis			
Effect on	Effects of	antiphase	¼ lag	antiphase	¼ lag	Speed	Cost
Prey (W_x)	$E_x^{(x)}$ - $E_u^{(x)}$	-/0	-/0	-/0/+	-	-0.44 ± 0.23	-0.43 ± 0.18
	$E_x^{(x)}$ - $E_v^{(x)}$	0/+	-/0/+	-/0/+	0/+	-0.34 ± 0.45	0.02 ± 0.20
	$E_y^{(x)}$ - $E_u^{(x)}$	**-/0**	**0/+**	**-**	**0/+**	**0.72 ± 0.05**	**0.70 ± 0.07**
	$E_y^{(x)}$ - $E_v^{(x)}$	-/0	-/0	-	0/+	0.29 ± 0.18	0.65 ± 0.18
	$E_u^{(x)}$ - $E_v^{(x)}$	-/0/+	-/0/+	-	0/+	0.09 ± 0.22	0.55 ± 0.11
Predator (W_y)	$E_x^{(y)}$ - $E_u^{(y)}$	**-**	+	**-**	+	**0.92 ± 0.02**	**0.80 ± 0.06**
	$E_x^{(y)}$ - $E_v^{(y)}$	-/0	+	-/0	0	0.67 ± 0.06	-0.07 ± 0.25
	$E_u^{(y)}$ - $E_v^{(y)}$	-/0/+	-/0/+	-/0	0	0.31 ± 0.36	0.52 ± 0.14

Appendix C: description of eco-evolutionary and component dynamics

Here we describe the eco-evolutionary dynamics of an example of antiphase cycles (Fig. C1a) and an example of ¼-lag cycles (Fig. C1b). The patterns of when prey and predator biomass increase or decrease can be understood more clearly by looking at the dynamics of the components resulting from the Geber method decomposition[14]. This describes how the change in the Malthusian fitness (per capita net growth rate; see eq. (4)) of the prey (W_x) and the predator (W_y) is affected by the change in the four variables:

$$\frac{dW_x}{dt} = \underbrace{E_x^{(x)}}_{\text{Effect of prey}} + \underbrace{E_y^{(x)}}_{\text{Effect of predator}} + \underbrace{E_u^{(x)}}_{\text{Effect of defense}} + \underbrace{E_v^{(x)}}_{\text{Effect of offense}}$$
$$\frac{dW_y}{dt} = \underbrace{E_x^{(y)}}_{\text{Effect of prey}} + \underbrace{E_y^{(y)}}_{\text{Effect of predator}} + \underbrace{E_u^{(y)}}_{\text{Effect of defense}} + \underbrace{E_v^{(y)}}_{\text{Effect of defense}} \qquad \text{(C1)}$$

C1. Antiphase cycles (Fig. C1a)

Generally, in antiphase cycles various strong changes happen simultaneously (e.g. an increase in prey biomass coinciding with a decrease in predator biomass, and with an increase in defense). As a result, component dynamics are generally strongly synchronized or anti-synchronized, giving rise to eco-evolutionary pattern where component effects partly or almost entirely cancel one another out (third and fourth panels).

t1 (*t* = 60): At the start of the cycle, both prey biomass and defense are low, while predator biomass and offense are high (top and second panel). Prey and predator fitness are both close to zero (bottom panel); little change is happening, and components are small and cancel each other out (third and fourth panels). At this point, offense is too high for increased defense to pay off: the difference in predation with a small increase in defense is not enough to offset the costs. However, because defense is low, offense slowly decreases.

t2 (*t* = 160): Offense finally reaches a low enough level for increased defense to become favourable. Defense now rapidly increases, resulting in a strong positive impact on the prey ($E_u^{(x)}$, third panel, dashed green line). This sudden increase in prey fitness (fifth and bottom row, green lines) causes prey biomass to rapidly increase as well. At the same time the increase in defense has a strong negative impact on the predator ($E_u^{(y)}$, fourth panel, dashed green line), which causes predator fitness to turn negative (bottom panel) and predator biomass to decline (top panel).

As defense increases, so does offense (second panel), impacting prey fitness negatively while having a positive impact on predator fitness ($E_v^{(x)}$ and $E_v^{(y)}$; third and fourth panels, dashed blue lines). The sudden increase in prey biomass also negatively impacts its own fitness (due to increased competition) while it increases predator fitness ($E_x^{(x)}$ and $Ex^{(y)}$; third and fourth panels, solid green lines). However, these effects are not strong enough to compensate for the impact of defense.

Appendix C

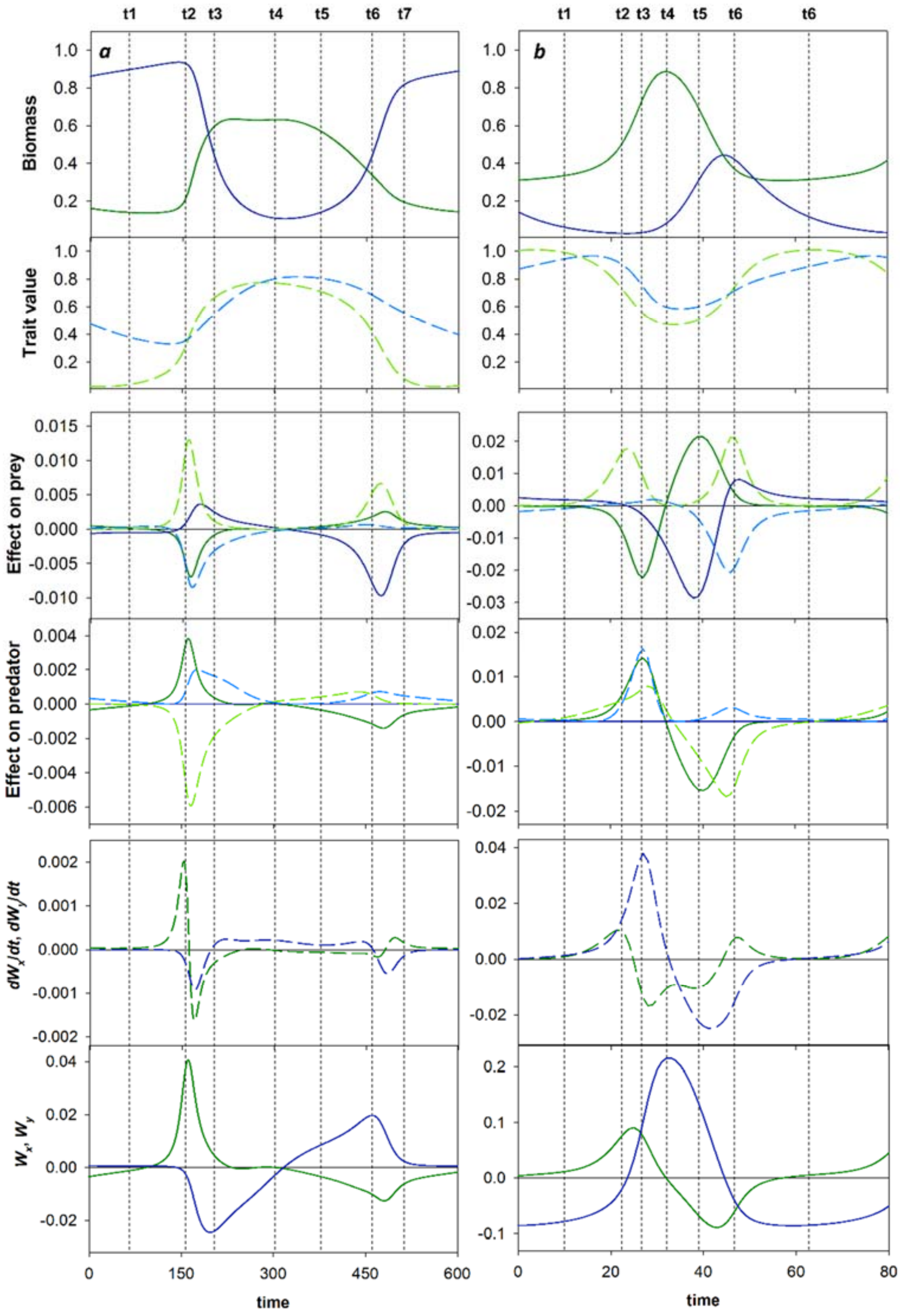

Figure C1: Eco-evolutionary dynamics (first and second row), dynamics of components resulting from Geber method decomposition (third and fourth row), dynamics of the total change in the per capita prey and predator growth rates, i.e. the sums of the components (fifth row), and the dynamics of the prey and predator per capita growth rates (sixth row). Top row: prey (solid green) and predator (solid blue) dynamics. Second row: defense (dashed green) and offense (dashed blue) dynamics. Third row: how the change in the prey net growth rate (W_x) is affected by changes in prey biomass ($E_x^{(x)}$, solid green), predator biomass ($E_y^{(x)}$, solid blue), defense ($E_u^{(x)}$, dashed green) and offense ($E_v^{(x)}$, dashed blue). Fourth row: how the change in the predator net growth rate (W_y) is affected by the changes in prey biomass ($E_x^{(y)}$), predator biomass ($E_y^{(y)}$), defense ($E_u^{(y)}$) and offense ($E_v^{(y)}$); colours have the same meaning as in the third row. Fifth row: sum of prey components (green) and predator components (blue), indicating the total change in the per capita net growth rates. Bottom row: per capita prey (green) and predator (blue) net growth rates. Parameter values are the same as those in Fig.

1a, 1b in the main text. Time is measured in time steps after the first 30,000 time steps of the simulation.

t3 (*t* = 200): Defense reaches a high value, resulting in high costs for increasing even further; the increase in defense slows down. Offense continues to increase, negatively impacting prey fitness ($E_V^{(x)}$, third panel; fifth panel, green line). Prey growth slows down and finally stops entirely; prey fitness approaches zero again (bottom panel) and prey biomass reaches a plateau. For the predator, the effect of offense now gradually begins to outweigh the effect of defense (fourth panel), and its fitness slowly increases (fifth panel, blue line), but not yet rapidly or strongly enough to halt the decline in predator biomass (bottom panel, blue line).

t4 (*t* = 300): The gradual increase in offense finally increases predator fitness enough to stop its decline, bringing it to its minimum. With predator biomass being so low, defense now becomes disfavoured and starts to fall, but initially changes are slow and all components are small (third and fourth panel). Prey and predator fitness are both close to zero (bottom panel) and their biomasses hold steady at their maximum and minimum, respectively (top panel).

t5 (*t* = 370): Defense starts to decrease more strongly. This has a positive impact on the predator, which starts to increase again; this in turn results in a negative impact on the prey, which starts to decline.

t6 (*t* = 455): The decline in prey biomass negatively impacts predator fitness; however, this is largely counterbalanced by the positive effects of the decline in defense and offense, and predator fitness increases to its maximum (bottom panel). Predator biomass increases rapidly (top panel). This has a strong negative impact on prey biomass, but again this is almost entirely compensated by the other changes: the decline in defense releases it from costs, while the decline in prey biomass itself releases it from competition. As a result, prey fitness decreases only slightly, and the decline in prey biomass continues as it was (top panel).

t7 (*t* = 510): Defense reaches a very low value and its decline slows substantially. The negative effect of $E_x^{(y)}$ (caused by the decline in prey biomass) on the predator now outweighs the positive effect of the decrease in offense $E_V^{(y)}$; predator growth slows down. This in turn positively impacts the prey (third panel); its decline slows down as well. From here on, little change happens for a significant amount of time. Defense is again at a low level while offense falls slowly; all component effects are small and almost entirely cancel out (fifth panel). The dynamics will remain almost stationary until offense becomes low enough for defense to increase (**t2**).

C2. ¼-lag cycles (Fig. C1b)

In contrast with antiphase cycles, component dynamics are here largely asynchronous (for the prey; third panel) or synchronized (for the predator; fourth panel). Note also that component effects are generally stronger here than in antiphase cycles, because changes of similar amplitude happen over a much shorter timespan (note the timescales on the x-axes in Fig. C1a and C1b).

t1 (*t* = 10): At the start of the predator-prey cycle, prey and predator biomass are both low (top panel) while defense and offense are high (second panel). Because predation pressure is low, defense starts to decline; after a short delay, offense follows.

t2 (*t* = 23): The decline in defense has a positive impact on the prey (releasing it from the high costs; $E_u^{(x)}$, third panel), and prey biomass increases. The declines in defense and offense and the increase in prey biomass all have a positive impact on the predator (fourth panel); the sum of these effects is strongly positive (fifth panel), and the predator stops declining and reaches its minimum (top panel).

t3 (*t* = 27): Prey biomass continues to strongly increase, while defense and offense continue to decrease. These changes all have a positive impact on the predator (fourth panel), and the total impact on the predator is very strongly positive (fifth panel). Predator fitness becomes positive (bottom panel) and predator biomass starts to increase. Meanwhile prey biomass approaches the carrying capacity, and competition starts to negatively affect it ($E_x^{(x)}$ becomes strongly negative, third panel). Prey growth begins to slow down.

t4 (*t* = 32): The increase in predator biomass slows down the decline in defense; in response to this, offense stops declining as well. All components become zero or close to zero for the predator (fourth panel); predator fitness is at its maximum (bottom panel), and predator biomass now increases rapidly. The strong negative impact on the prey (third panel) causes the start of the decline in prey biomass (top panel).

t5 (*t* = 39): Prey biomass declines while predator biomass is still strongly increasing. The increase in predator biomass has a strong negative impact on the prey; while its own decline has a substantial positive impact, this is outweighed by the negative impact of the predator (third panel, solid lines; fifth panel, green line). Prey fitness declines further (bottom panel) and the decline in prey biomass becomes even more rapid (top panel). The decline in prey biomass, combined with the increase in defense, results in a strong negative impact on predator fitness (fourth panel); predator growth slows down (top panel).

t6 (*t* = 46): Defense and offense continue to increase while prey biomass continues to fall; the continued negative impacts on the predator (fourth panel) finally cause predator fitness to turn negative (bottom panel) and causes predator biomass to start declining again (top panel). This in turn has a positive impact on the prey (third panel), slowing down its decline.

t7 (*t* = 63): The predator continues to decline towards its minimum; this has a small positive impact on the prey, and gradually prey biomass starts increasing again (**t1**).

Appendix D: Effects of secondary component correlations

Only one of the component correlations we analyzed was found to be strongly associated with the predator-prey phase relationship in both the speed analysis and the cost analysis ($E_x^{(y)} – E_u^{(y)}$; that is, the correlation between the effect of changing prey biomass and of changing defense on the net per capita predator growth rate W_y; Fig. D1, top row). However, in addition to this, several other component correlations were found to be associated with the predator-prey phase relationship in only one of the analysis types. One component correlation stands out in the speed analysis, but has no association with the phase relationship at all in the cost analysis ($E_x^{(x)} – E_v^{(x)}$; Table 2, Fig. D2, Fig. D3). The reverse is true for two component correlations that were not associated with the phase relationship in the speed analysis, but were associated in the cost analysis ($E_y^{(x)} – E_u^{(x)}$ and $E_u^{(x)} – E_v^{(x)}$; Table 2, Fig. D2, Fig. D3). Here we explain what underlies these secondary component correlations, why their associations are restricted to only one analysis type, and why they have far less explanatory power than the main result.

¼-lag cycles in the speed analysis are a result of rapid predator adaptation, whereas ¼-lag cycles in the cost analysis are a result of a high costliness of defense. While the predator-prey phase relationship is the same in both cases, the eco-evolutionary dynamics in these two scenarios are very different (compare Fig. 1b, 1c in main text). The most pronounced difference is that in the case of high costs for defense, the amplitude of trait dynamics is strongly dampened (see Fig.1c). Thus, changes in one trait do not strongly affect the ecological dynamics, or the dynamics of the other trait. Instead, both traits respond mainly to the ecological dynamics: offense increases when prey biomass is low, and vice versa; similarly, defense increases when predator biomass is high, and vice versa. These defense dynamics are consistent with those found in an experimental study on defense with non-adaptive predators[15]; however, in contrast to this study, in our model this does not result in antiphase cycles because the oscillations in defense have only a small effect.

In summary, in the case of a high costliness for defense (causing ¼-lag cycles in the cost analysis), the dynamics of the two traits become decoupled. In contrast, rapid predator adaptation (causing ¼-lag cycles in the speed analysis) results in a strong arms race between offense and defense, causing the two traits to cycle nearly in phase. This in turn causes some of the component correlations for ¼-lag cycles to differ between the speed analysis and the cost analysis, resulting in associations that are restricted to one analysis type.

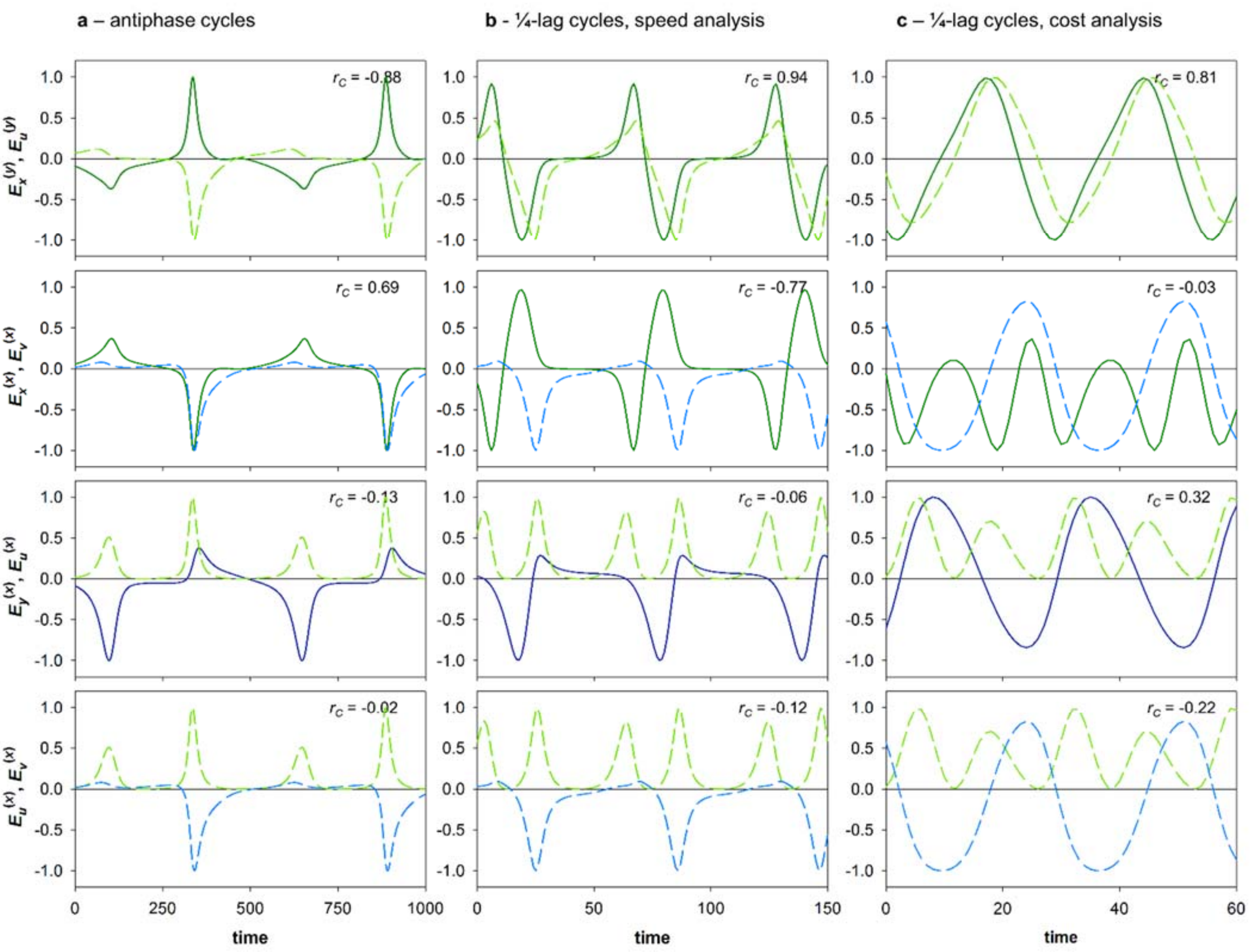

Figure D1: Component dynamics and component correlations for the different types of eco-evolutionary cycles. D1a-c correspond to Fig. 1a-c in the main Results. Lines show component effects, standardized with respect to their absolute maximum over time, so their range is between -1 and 1: effect of prey (E_x, solid green), effect of predator (E_y, solid blue), effect of defense (E_u, dashed green) and effect of offense (E_v, dashed blue), impacting W_y (top row) or W_x (bottom three rows). For each component pair, the component correlation r_C is shown for these specific simulation runs. (a): antiphase cycles, standard parameters for speed analysis (see Table 1) with $G_x = G_y = 10^{-2}$. (b) ¼-lag cycles caused by rapid predator adaptation, standard parameters for speed analysis with G_x 10^{-1}, $G_y = 10^{-0.9}$. (c) ¼-lag cycles caused by high cost of defense, standard parameters for cost analysis with $c_x = 5$, $h = 2$.

Secondary association found only in the speed analysis

In the speed analysis, one secondary component correlation stands out as strongly associated with the phase relationship: $E_x^{(x)} - E_v^{(x)}$ (Table 2; Fig. D1, second row; Fig. D2, second column). This component correlation is positive in the case of antiphase cycles (Fig. D1a, second row), and negative in the case of ¼-lag cycles (Fig. D1b, second row; summarized in Fig. D2, second column). In the case of antiphase cycles, changes in prey biomass and in offense always affect prey fitness in the same direction. This means that an increase in prey biomass (negatively impacting prey fitness) coincides with an increase in offense (also negatively impacting prey fitness), and vice versa; i.e. prey biomass and offense

cycle in phase. The reverse is true for ¼-lag cycles, where prey biomass and offense cycle out of phase (Fig. 1a, 1b).

Because offense cycles nearly in phase with defense, lagging only slightly behind (Fig. 1a, 1b), this association only reflects the main result: that prey biomass and defense cycling in phase generates antiphase predator-prey cycles, while prey biomass and defense cycling in antiphase generates ¼-lag predator-prey cycles. The fact that this association is not present in the cost analysis confirms this interpretation: in ¼-lag cycles in the cost analysis, the dynamics of offense do not closely follow those of defense (Fig. D1c, second row), and this component correlation has no association with the phase relationship (Table 2; Fig. D3, second column). Thus, it appears unlikely that this component correlation is driving the predator-prey phase relationship.

Secondary associations found only in the cost analysis

Two further component correlations are only associated with the phase relationship in the cost analysis: the correlations between the effects of defense and offense on the prey ($E_u^{(x)}$ - $E_v^{(x)}$) and between the effects of predator biomass and defense on the prey ($E_y^{(x)}$ - $E_u^{(x)}$) (Table 2; Fig. D3). Both associations reflect the difference in the trait dynamics between antiphase and ¼-lag cycles in the cost analysis. In the case of antiphase cycles, trait dynamics are strongly driven by the arms race between defense and offense; in the case of ¼-lag cycles, unlike the ¼-lag cycles in the speed analysis, the dynamics of the two traits respond to the ecological dynamics but not to each other. Thus, the presence of the trait arms race in antiphase cycles, and the absence of it in ¼-lag cycles, is the underlying cause for these two associations.

As a result of this, in antiphase cycles, the effect of defense ($E_u^{(x)}$) is largely decoupled from that of predator biomass ($E_y^{(x)}$), while the two effects are temporally linked in ¼-lag cycles. This results in a positive $E_y^{(x)}$ - $E_u^{(x)}$ component correlation in the case of ¼-lag cycles (Fig. D1c, third row), while no correlation is present in the case of antiphase cycles (Fig. D1a, third row) or ¼-lag cycles in the speed analysis (Fig. D1b, third row).

Likewise, the effects of defense and offense are strongly temporally linked in antiphase cycles, and the arms race causes the dynamics of the two traits to be nearly in phase. Because $E_u^{(x)}$ is always positive, both increases and decreases in defense cause a positive peak in $E_u^{(x)}$. One of these peaks will coincide with a negative peak in $E_v^{(x)}$, reflecting the simultaneous increase in offense and defense, while the other coincides with a (weaker) positive peak in $E_v^{(x)}$, and the overall result is no correlation between the two components (Fig. D1a-b, bottom row; Fig. D2, fourth column). Because defense and offense dynamics are decoupled in the ¼-lag cycles in the cost analysis, a negative component correlation appears (Fig. D1c, bottom row; Fig. D3, fourth column), but this looks like an artefact rather than a result with predictive power.

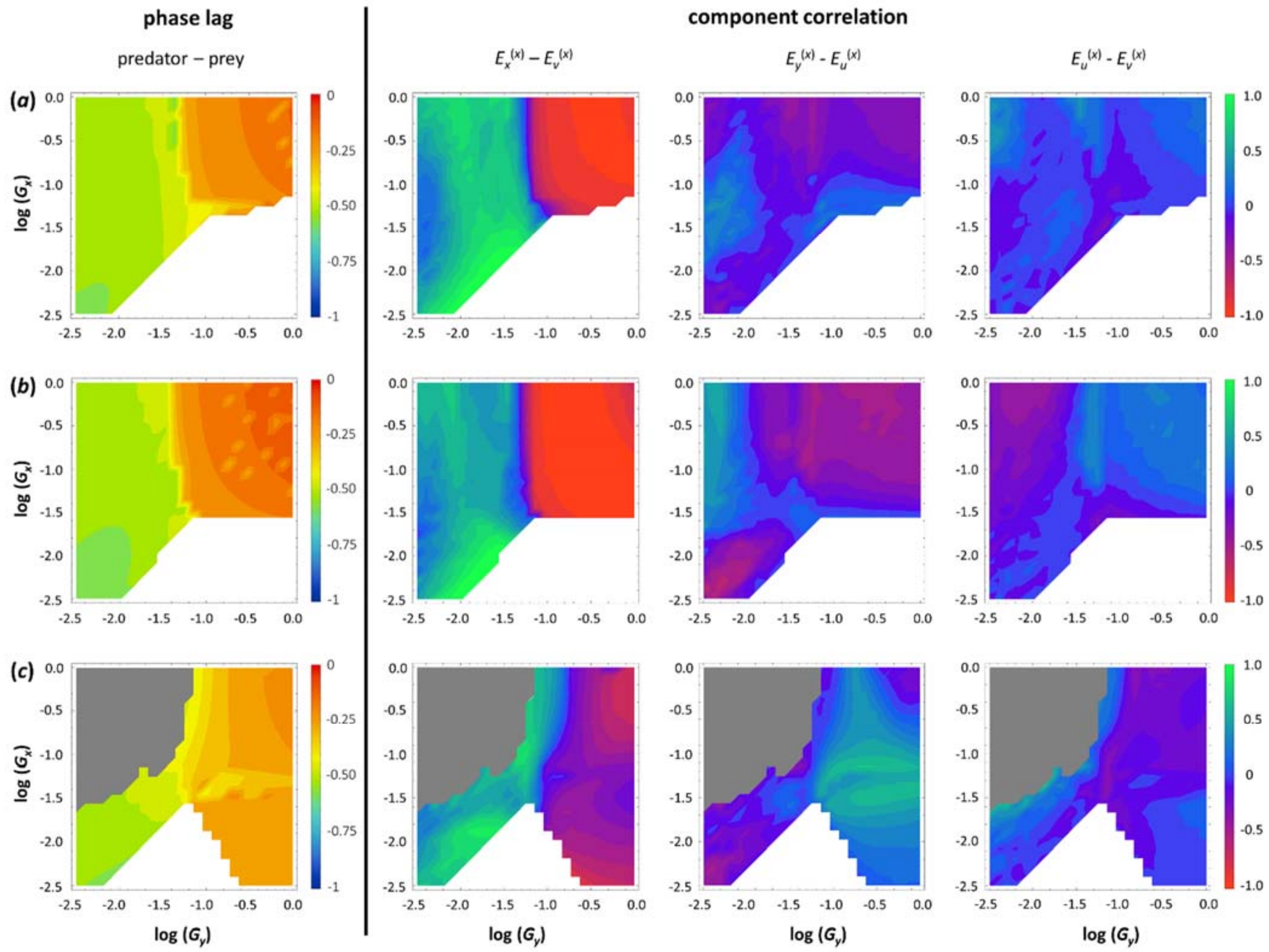

Figure D2: Effect of the speed of adaptation in prey and predator on the predator-prey phase relationship (left) and on three component correlations. Second column: component correlation $E_x^{(x)} - E_v^{(x)}$ (the effects of prey biomass and offense on the prey), showing a positive correlation in the case of antiphase cycles and a negative correlation in the case of ¼-lag cycles. Third and fourth column: $E_y^{(x)} - E_u^{(x)}$ (effects of predator biomass and defense on the prey) and $E_u^{(x)} - E_v^{(x)}$ (effects of defense and offense on the prey), respectively, showing no association with the phase relationship. White: stable equilibrium; grey: extinction of predator. (a) Standard parameters (see Table 1); (b) standard parameters except $K = 2$ (higher carrying capacity); (c) standard parameters except $h = 2$ (long handling time).

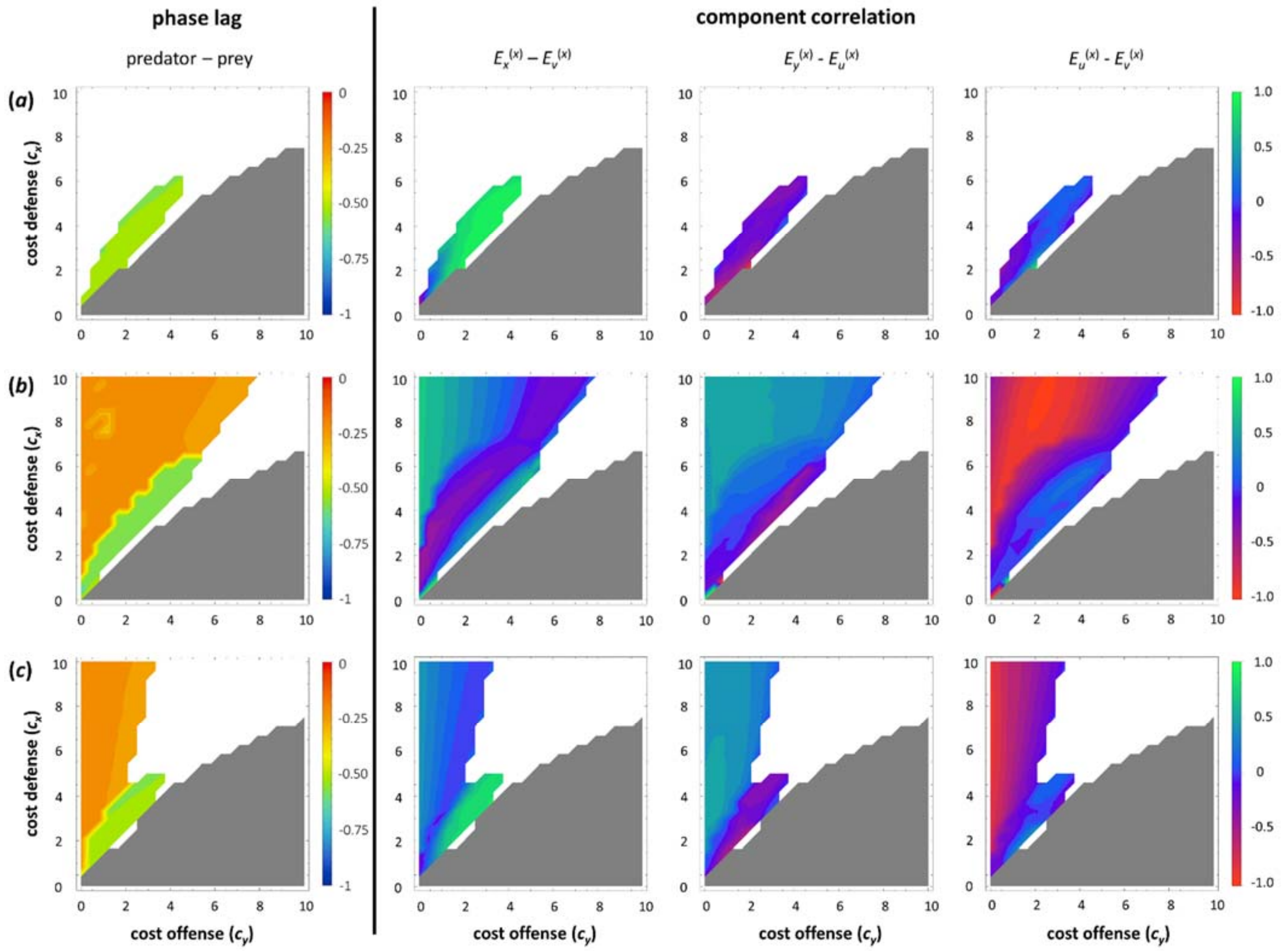

Figure D3: Effect of costliness of defense and offense on the predator-prey phase relationships (left) and three component correlations. Second column: component correlation $E_x^{(x)} - E_v^{(x)}$ (the effects of prey biomass and offense on the prey), showing no consistent association with the phase relationship in the cost analysis. Third column: $E_y^{(x)} - E_u^{(x)}$ (effects of predator biomass and defense on the prey), showing a weak negative correlation in the case of antiphase cycles and a weak positive correlation in the case of ¼-lag cycles. Fourth column: $E_u^{(x)} - E_v^{(x)}$ (effects of defense and offense on the prey), showing no correlation in the case of antiphase cycles and negative correlation in the case of ¼-lag cycles. White: stable equilibrium; grey: extinction of predator. (a) Standard parameters for cost analysis; (b) standard parameters except $K = 2$ (high carrying capacity); (c) standard parameters except $h = 2$ (long handling time).

Appendix E: Detailed analysis of the transition from antiphase to ¼-lag cycles

In both the speed analysis and the cost analysis, the predator-prey phase lag switches suddenly and sharply from antiphase to ¼-lag cycles as the speed of predator adaptation (speed analysis; Fig. E1a) or the cost of defense (cost analysis; Fig. E1b) increases. We describe in detail how this transition occurs in both cases below.

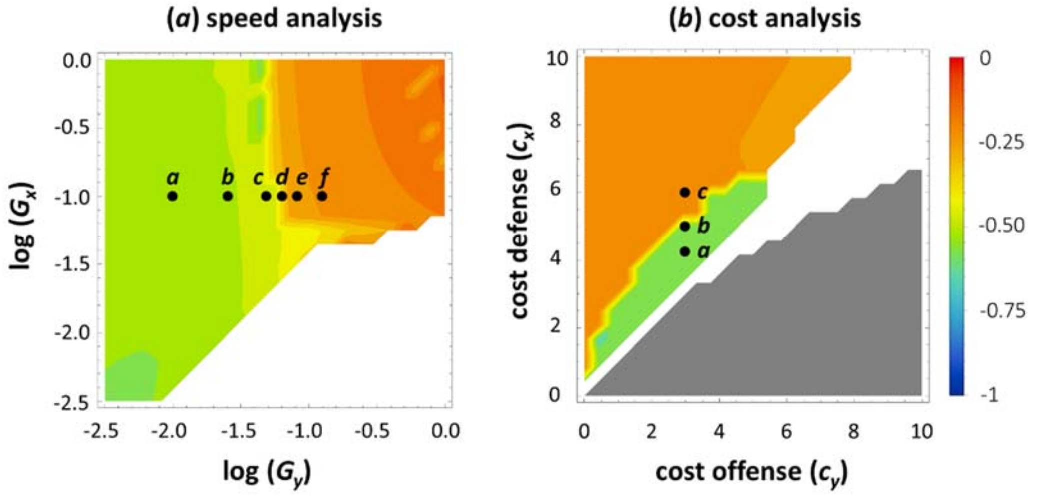

Figure E1: Predator-prey phase relationships of two simulation sets shown in the main text: (a) standard parameters for speed analysis (Table 1; cf. Fig. 2a in main text); a-f correspond to dynamics shown in in Fig. E2. (b) Standard parameters for cost analysis, except $K = 2$ (Table 1; cf. Fig. 3b in main text); a-c correspond to the parameter values used in Fig. E3.

Transition from antiphase to ¼-lag cycles with increasing speed of predator adaptation

In the speed analysis, a narrow transition range between antiphase and ¼-lag cycles can be observed, where the phase lag is intermediate (the yellow and yellowish areas in Fig. E1a). Although this range is generally very narrow (Fig. 2 in main text), the transition from antiphase to ¼-lag is gradual (Fig. E2).

As described in the Results, the peak in prey biomass may be generated by either an increase in defense (for antiphase cycles) or a decrease in defense (for ¼-lag cycles). This is because both the increase and the decrease in defense always have a positive impact on prey growth; the relative strength of these two positive effects determines whether the predator-prey phase relationship is antiphase or ¼-lag. When the positive impact of the increase in defense is very strong, the resulting release from predation allows the prey to rapidly grow to high biomass (Fig. 1a; Fig. E2a): prey biomass and defense then cycle synchronously, resulting in antiphase cycles. In contrast, when the positive impact of the decrease in defense is strong, the release from the costs of defense allows the prey peak to occur (Fig. 1b; Fig. E2f): prey biomass and defense then cycle anti-synchronously, resulting in ¼-lag cycles.

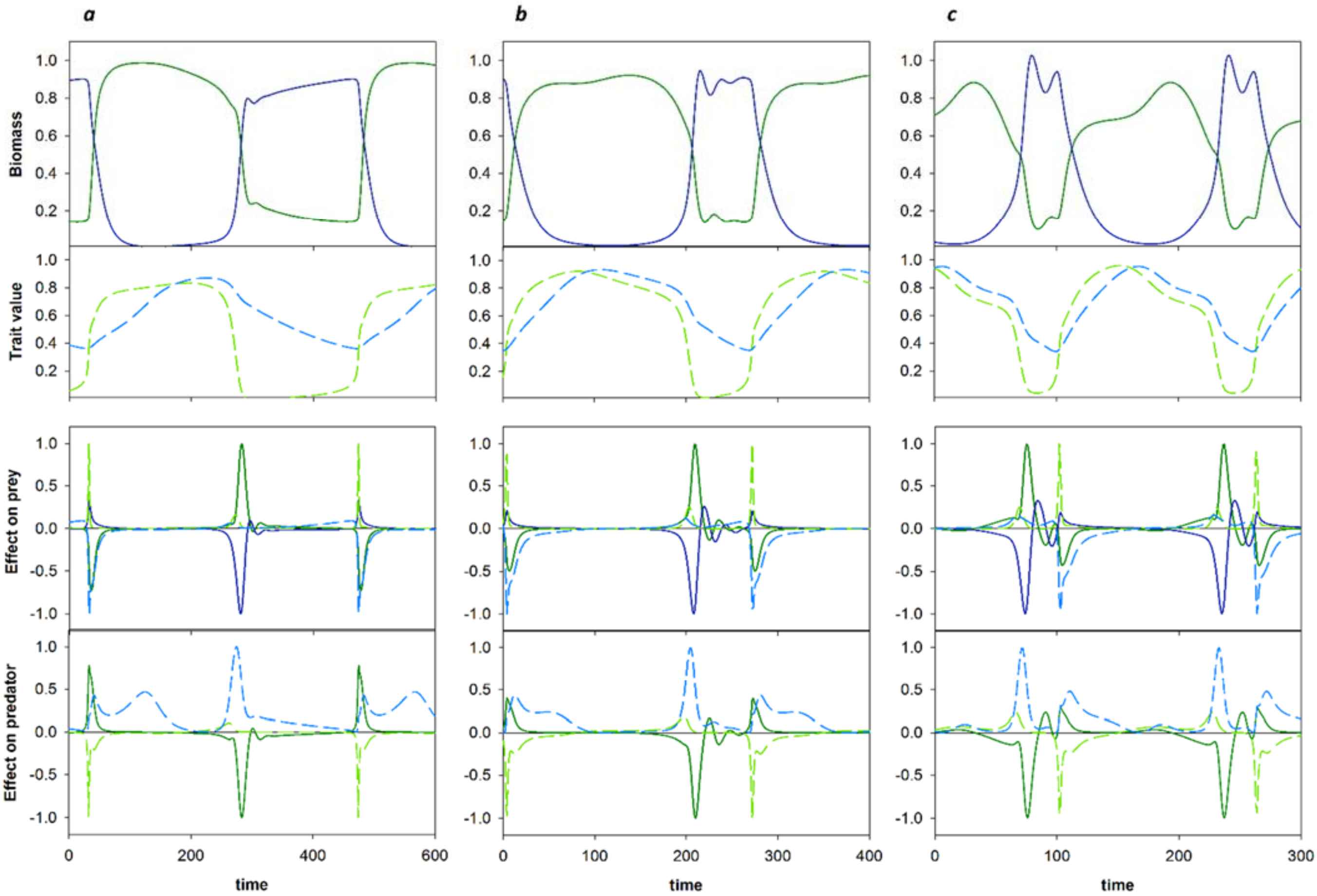

Figure E2: Eco-evolutionary dynamics and dynamics of components resulting from Geber method decomposition, for the parameter combinations shown in Fig. E1a (increasing the speed of predator adaptation). Top row: prey (solid green) and predator (solid blue) dynamics. Second row: defense (dashed green) and offence (dashed blue) dynamics. Third row: how the change in the prey growth rate (W_x) is affected by changes in prey biomass ($E_x^{(x)}$, solid green), predator biomass ($E_y^{(x)}$, solid blue), defense ($E_u^{(x)}$, dashed green) and offence ($E_v^{(x)}$, dashed blue). Bottom row: how the change in the predator growth rate (W_y) is affected by the changes in prey biomass ($E_x^{(y)}$), predator biomass ($E_y^{(y)}$), defense ($E_u^{(y)}$) and offence ($E_v^{(y)}$). In all, $G_x = 10^{-1}$. (a): $G_y = 10^{-2}$; (b) $G_y = 10^{-1.6}$; (c): $G_y = 10^{-1.3}$. Time is measured in time steps after the first 30,000 time steps of the simulation. Component effects are standardized with respect to their absolute maxima over time, so the range is between -1 and 1.

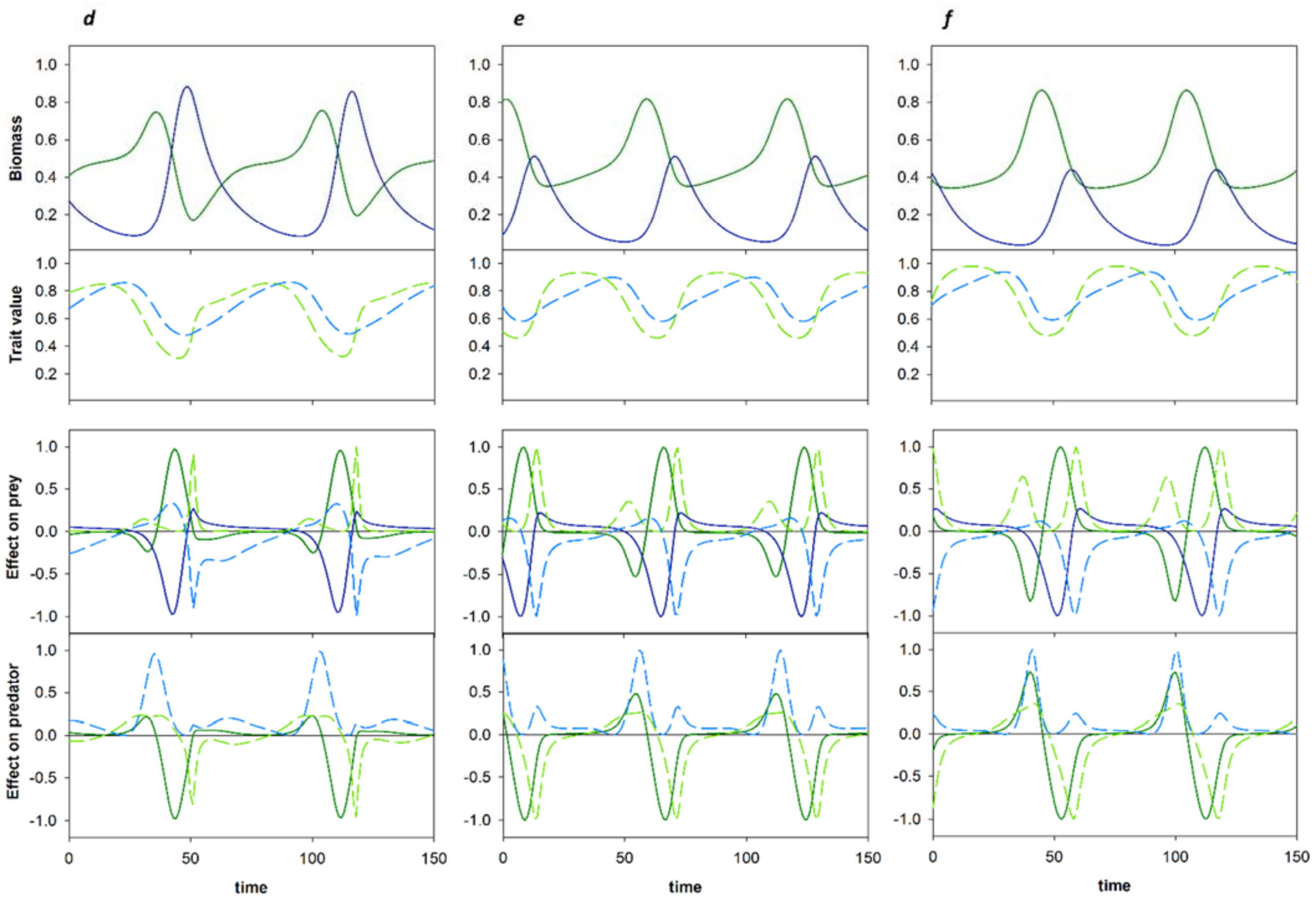

Figure E2 (continued): Eco-evolutionary dynamics and dynamics of components resulting from Geber method decomposition, for the parameter combinations shown in Fig. E1a. (d) $G_y = 10^{-1.2}$; (e) $G_y = 10^{-1.1}$; (d) $G_y = 10^{-1}$

The two scenarios outlined in the paragraph above are the extremes. As the speed of predator adaptation increases (Fig. E2a-f), there is a gradual shift between the relative importance of the increase and decrease in defense. When predator adaptation is slow, the increase of defense has by far the strongest impact: the increase in prey biomass is entirely associated with the increase in defense (Fig. E2a). As the speed of predator adaptation increases, defense is driven to higher levels (Fig. E2b-c), resulting in higher costs of defense. Thus, when defense starts to decrease, this has a more positive effect on the prey, which generates a second increase in prey biomass. Initially this second peak is very small (Fig. E2b), but it becomes more pronounced as the speed of predator adaptation increases, while the peak associated with the increase in defense becomes gradually less pronounced (Fig. E2b-e). Eventually the first peak disappears entirely (Fig. E2f), leaving only the second one, resulting in ¼-lag cycles. At the transition, when the increases in prey biomass caused by increasing and decreasing defense are of similar magnitude, phase lags between antiphase and ¼-lag cycles may be found (Fig. E2d).

The dynamics of the component $E_u^{(x)}$ (the impact of defense on the prey) show this gradual transition: while increasing defense is always associated with a stronger positive impact than decreasing defense, the relative magnitudes of these two positive impacts shifts as the speed of predator adaptation increases (Fig. E2a-f, third row).

Appendix E

Transition from antiphase to ¼-lag cycles with increasing costliness of defense

In the cost analysis, predator-prey dynamics are always classified as either antiphase cycles or ¼-lag cycles; although the transition from one type of dynamic to the other is again gradual, no intermediate phase lags are found (Fig. E2b). Instead, during the transition from antiphase to ¼-lag dynamics, complex oscillations are found: slower antiphase predator-prey cycles resulting from trait dynamics are superimposed on rapid ¼-lag predator-prey cycles (Fig. E3). Due to the way the predator-prey phase lag is calculated (i.e. using the dominant frequency of the Fourier spectrum), all phase lags are classified as either antiphase or ¼-lag cycles even if both signals are present, depending on the relative strength of the antiphase and ¼-lag signals.

Antiphase cycles are found under the condition that the costliness of defense is relatively low, i.e. at the lower range of c_x where predator-prey cycles are found at all (Fig. E1b; Fig. 3 in main text). Due to the low costs, prey can evolve a high level of defense (Fig. E3a). Predators are forced to follow, and the costs they incur (reduced conversion efficiency) dampens ¼-lag cycles entirely or almost entirely, resulting in clear antiphase predator-prey cycles (Fig. E3a). As the costliness of defense increases, the maximum and average levels of defense decrease; as a result, the level of offense is lowered as well, and dynamics of both traits become smaller in amplitude (Fig. E3b-c). Thus, the impact of trait changes on predator-prey dynamics becomes weaker, while the lowered costs for the predator result in stronger ¼-lag cycles (Fig. E3b-c). Eventually, at high costliness of defense, trait changes become very small and ¼-lag cycles very strong (Fig. 1c in main text).

The transition from antiphase to ¼-lag cycles happens when the antiphase and ¼-lag signals are equally strong (Fig. E3b). Here, due to the combination of slow trait dynamics (especially of offense; Fig. E3b) and rapid ecological dynamics, the dynamics of the effective biomass can become very complex, with variations in period length as well as amplitude. This causes difficulties in calculating, or even defining, the phase relationships between predator and effective prey, and between actual and effective prey, as the Fourier transform only gives meaningful results for constant period lengths. This causes the anomalous phase relationships that are sometimes found at the transition (see especially Fig. 3b in main text).

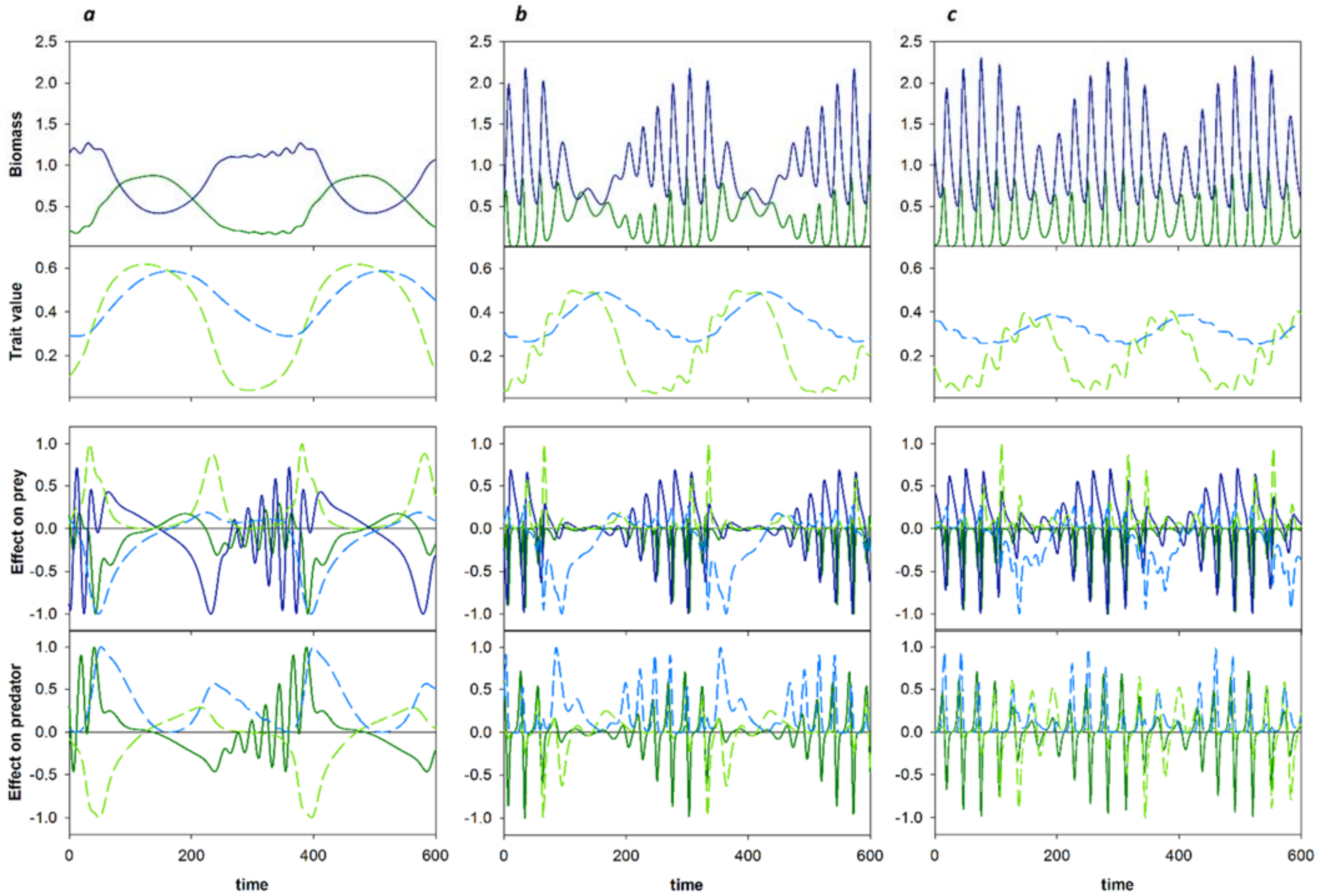

Figure E3: Eco-evolutionary dynamics and dynamics of components resulting from Geber method decomposition, for the parameter combinations shown in Fig. E1b (increasing the costliness of defense). In all, c_y =3.0. (a) c_x = 4.0; the main predator-prey dynamic is slow antiphase cycles, but small ¼-lag oscillations occur when offense is higher than defense. (b) c_x = 5.0; the strengths of the antiphase and ¼-lag signals are nearly equal, and complex predator-prey oscillations are found. (c) c_x = 6.0; the ¼-lag signal is clearly stronger, though the impact of the trait dynamics is visible in the variable amplitudes of predator-prey oscillations.

References

1. Abrams, P. A. The evolution of predator-prey interactions: Theory and evidence. *Annu. Rev. Ecol. Syst.* **31**, 79-105 (2000).

2. Saloniemi, I. A coevolutionary predator-prey model with quantitative characters. *Am. Nat.* **141**, 880-896 (1993).

3. Abrams, P. A. & Matsuda, H. Fitness minimization and dynamic instability as a consequence of predator-prey coevolution. *Evol. Ecol.* **11**, 1-20 (1997).

4. Sasaki, A. & Godfray, H. C. J. A model for the coevolution of resistance and virulence in coupled host-parasitoid interactions. *Proc. R. Soc. B-Biol. Sci.* **266**, 455-463 (1999).

5. Nuismer, S. L., Ridenhour, B. J. & Oswald, B. P. Antagonistic coevolution mediated by phenotypic differences between quantitative traits. *Evolution* **61**, 1823-1834 (2007).

6. Yoshida, T., Jones, L. E., Ellner, S. P., Fussmann, G. F. & Hairston, N. G. Rapid evolution drives ecological dynamics in a predator-prey system. *Nature* **424**, 303-306 (2003).

7. Becks, L., Ellner, S. P., Jones, L. E. & Hairston, N. G., Jr. Reduction of adaptive genetic diversity radically alters eco-evolutionary community dynamics. *Ecol. Lett.* **13**, 989-997 (2010).

8. Pahlow, M. & Prowe, A. E. F. Model of optimal current feeding in zooplankton. *Mar. Ecol. Prog. Ser.* **403**, 129-144 (2010).

9. Kiørboe, T. How zooplankton feed: mechanisms, traits and trade-offs. *Biol. Rev.* **86**, 311-339 (2011).

10. Mougi, A. & Iwasa, Y. Unique coevolutionary dynamics in a predator-prey system. *J. Theor. Biol.* **277**, 83-89 (2011).

11. Mougi, A. Predator-prey coevolution driven by size selective predation can cause anti-synchronized and cryptic population dynamics. *Theor. Popul. Biol.* **81**, 113-118 (2012).

12. Abrams, P. A. Modelling the adaptive dynamics of traits involved in inter- and intraspecific interactions: An assessment of three methods. *Ecol. Lett.* **4**, 166-175 (2001).

13. Tien, R. J. & Ellner, S. P. Variable cost of prey defense and coevolution in predator-prey systems. *Ecol. Monogr.* **82**, 491-504 (2012).

14. Hairston, N. G., Ellner, S. P., Geber, M. A., Yoshida, T. & Fox, J. A. Rapid evolution and the convergence of ecological and evolutionary time. *Ecol. Lett.* **8**, 1114-1127 (2005).

15. Becks, L., Ellner, S. P., Jones, L. E. & Hairston, N. G., Jr. The functional genomics of an eco-evolutionary feedback loop: linking gene expression, trait evolution, and community dynamics. *Ecol. Lett.* **15**, 492-501 (2012).
